# Combined FOLFOX4 with all-trans retinoic acid versus FOLFOX4 with placebo in treatment of advanced hepatocellular carcinoma with extrahepatic metastasis: a randomized, double-blind comparative study

**DOI:** 10.1038/s41392-023-01604-3

**Published:** 2023-09-27

**Authors:** Juxian Sun, Feifei Mao, Chang Liu, Fan Zhang, Dafeng Jiang, Weixing Guo, Lei Huo, Liping Zhou, Wan Yee Lau, Jie Shi, Shuqun Cheng

**Affiliations:** 1 Department of Hepatic Surgery VI, Third Affiliated Hospital of Naval Medical University, Shanghai, China; 2 Tongji University Cancer Center, Shanghai Tenth People’s Hospital, School of Medicine, Tongji University, Shanghai, China; 3 Department of General Surgery, Fujian Cancer Hospital, Fujian Medical University, Fuzhou, China; 4 Department of Oncology, Zhejiang Sian International Hospital, Jiaxing, China; 5 Department of Radiology, Third Affiliated Hospital of Naval Medical University, Shanghai, China; 6 Faculty of Medicine, The Chinese University of Hong Kong, Prince of Wales Hospital, Shatin, New Territories, Hong Kong SAR, China

**Keywords:** Gastrointestinal cancer, Cancer therapy

## Abstract

The majority of hepatocellular carcinoma (HCC) cases are diagnosed at an advanced stage. Currently, there are only a few therapeutic methods available for patients with advanced HCC and extrahepatic metastasis (EHM). Systemic chemotherapy, such as FOLFOX4 (infusions of fluorouracil, leucovorin, and oxaliplatin), has been reported for treating advanced HCC with EHM, but its effectiveness is very poor. In this randomized, double-blind, placebo-controlled study, we aimed to assess the efficacy and safety of FOLFOX4 with all-trans-retinoic acid (ATRA) as a palliative treatment for HCC patients with EHM, compared to FOLFOX4 with a placebo. The primary endpoint was overall survival (OS), and subsequently, an exploratory model was developed based on bioinformatics to predict the efficacy of FOLFOX4-ATRA treatment. A total of 108 patients were randomly assigned in a 1:1 ratio to receive either FOLFOX4-ATRA or FOLFOX4-placebo. The intention-to-treat (ITT) population showed a median OS of 16.2 months for the FOLFOX4-ATRA group, compared with 10.7 months for the FOLFOX4-placebo group (HR 0.56, 95% CI 0.33–0.93; *p* = 0.025). The median progression-free survival (PFS) was 7.1 months for the FOLFOX4-ATRA group and 4.2 months for the FOLFOX4-placebo group (HR 0.62, 95% CI 0.41–0.94; *p* = 0.024). A panel of proteins with unique upregulation during complete response (CR) (SOD3, TTR, SSC5D, GP5, IGKV1D-33) and partial response (PR) (TGFB1, GSS, IGHV5-10-1) effectively predicted CR and PR in patients treated with FOLFOX4-ATRA, as compared to FOLFOX4-placebo. The results suggest that FOLFOX4-ATRA is a safe and effective treatment for patients with advanced HCC and EHM in eastern China.

## Introduction

Hepatocellular carcinoma (HCC) is the sixth most common malignancy, with more than 626,000 new cases per year, and the third leading cause of cancer-related deaths worldwide.^1,2^ However, potentially curative therapies can only be applied in approximately 15% of these patients^3^ and the 5-year recurrence rate after treatment with curative intent can be as high as 75%.^4,5^ Extrahepatic metastasis (EHM) can occur either at the time of first diagnosis or at recurrence, with a combined incidence of EHM of 25–56%.^6–8^ Once EHM occurs, locoregional treatment, either alone or in combination, can no longer be used, and the prognosis will be extremely poor.

In recent years, systematic treatment of advanced HCC has made great progress and includes targeted therapy, immune checkpoint inhibitor (ICI) therapy, and intravenous chemotherapy. The updated results of the IMbrave150 trial^9^ revealed that the median overall survival (OS) and progression-free survival (PFS) were 19.2 months and 6.9 months with atezolizumab plus bevacizumab (T + A) for advanced HCC patients. The objective response rate (ORR) was 30%, and the complete response (CR) rate was 8%. However, only 63% of the HCC patients in the atezolizumab plus bevacizumab group had EHM, and 17% were Barcelona Clinic Liver Cancer (BCLC) stage A/B. Unlike the combination of targeted therapy and ICIs in the IMbrave150 study, the phase III HIMALAYA trial^10^ used dual ICIs (tremelimumab added to durvalumab). The results showed that the median OS of tremelimumab added to durvalumab was 16.4 months, which was significantly higher than the 13.8 months of the sorafenib group. The ORR was 20.3%, of which the CR rate was 3%. Similar to the IMbrave150 trial, the HIMALAYA trial was also not specifically designed to evaluate advanced HCC with EHM, and we cannot accurately determine the effect of atezolizumab plus bevacizumab or tremelimumab added to durvalumab on advanced HCC with EHM until further disclosure of the subgroup analysis data, which may reduce the reference value of these two studies for the treatment of advanced HCC with EHM. A retrospective multicenter study^11^ on the use of apatinib to treat HCC patients with pulmonary metastasis showed a median PFS of 5 months, with an ORR of 22.0%. Above all, in addition to its costs and adverse events (AEs), its low efficiency is still the most important factor limiting the clinical application of targeted therapy and ICIs for advanced HCC with EHM.

Chemotherapy for HCC, especially in Asia, is still being studied, although HCC is recognized as one of the most drug-resistant tumors, and many chemotherapy drugs or regimens^12^ have been indicated to be ineffective for HCC or have intolerable toxicity, and some of these regimes include doxorubicin, cisplatin/interferon alpha-2b/doxorubicin/fluorouracil (PIAF), and gemcitabine plus cisplatin/oxaliplatin. The encouraging results of two phase III studies conducted by Qin et al. ^13^ and Lyn et al. ^14^ revealed that FOLFOX4 (oxaliplatin +5-fluorouracil/leucovorin) may confer some benefits to advanced HCC patients in Asia, with an acceptable safety profile. Although the FOLFOX4 regimen has gained approval for the treatment of advanced HCC in China and has served as an empiric treatment option by some physicians in the United States,^15–17^ it must be acknowledged that the benefits that advanced HCC patients receive from current chemotherapy regimens are still very limited, and the incidence of AEs is also relatively high. Additionally, we consider that the prognosis of advanced HCC patients is highly heterogeneous and needs to be reclassified for treatment.^18^ Although the above well-designed studies obtained positive results, the heterogeneous enrolled population would make the results difficult to be interpreted. Future studies that take these heterogeneities into account are needed.

All-trans retinoic acid (ATRA), a naturally occurring derivative of vitamin A, is a known strong differentiation inducer. ATRA can activate the heterodimer retinoic acid receptor (RAR)/retinoic X receptor (RXR) by binding to RARs and then binds to the retinoic acid response element (RARE) of the upstream enhancer or promoter of the target gene to activate the target gene. ATRA was first used in the treatment of hematological tumors and has become the standard frontline treatment for acute granulocytic leukemia (APL).^19^ Its application to solid tumors is also being tried,^20,21^ and our previous studies showed that ATRA potentiates the chemotherapeutic effect of cisplatin/oxaliplatin by inducing the differentiation of liver cancer-initiating cells and eliciting tumor cell cycle arrest.^22,23^ In a retrospective study on advanced HCC patients reported by our group,^24^ the combined use of FOLFOX4 and ATRA resulted in an ORR of 22.8%. When compared with FOLFOX4 alone, FOLFOX4 combined with ATRA resulted in significantly better median overall survival (14.9 vs. 8.2 months, *p* < 0.001), and subgroup analysis showed that FOLFOX4 combined with ATRA seemed to be more effective for EHM than for hepatic tumors. This prospective double-blinded randomized study was conducted to determine whether palliative systemic chemotherapy with FOLFOX4-ATRA can result in a significant survival benefit compared to FOLFOX4-placebo in treating advanced HCC patients with EHM. This study also aimed to develop the specific response markers and potential targets for predicting the efficacy of FOLFOX4-ATRA treatment based on the proteomics and bioinformatics obtained in this study.

## Patients and methods

### Study design

A randomized, placebo-controlled, double-blind, multicenter clinical trial was performed on FOLFOX4-ATRA versus FOLFOX4-placebo in advanced HCC patients with EHM. Written informed consent of each participant was obtained. Randomization was performed by an independent statistician using SAS 9.4 SID to generate randomization. Eligible subjects who were enrolled in the study received a unique subject identification number, and they were randomly assigned to the FOLFOX4-ATRA or FOLFOX4-placebo treatment group at a ratio of 1:1. ATRA tablets and placebos had the same appearance, packaging, and administration schedules. Both the subjects and investigators were blinded to the treatments until emergency unblinding or after database lock.

This trial was registered on Chinese Clinical Trail Registry (ID: ChiCTR-IIR-17012916, www.chictr.org.cn) on 9 October 2017.

### Patient eligibility

As mentioned in our previous study,^24^ patients eligible for this trial had unresectable HCC with measurable EHM located in the lung, abdomen, or bone according to mRECIST (≥1 cm on spiral CT or MRI), an Eastern Cooperative Oncology Group performance status grade of 1 or less, Child‒Pugh class A, a life expectancy of more than 3 months and adequate organ function. Previous treatments, including chemotherapeutic agents, immunotherapy, or targeted therapy, had to be completed 4 weeks before randomization. The exclusion criteria included allergy to study drugs, a history of FOLFOX4 treatment, central nervous system (CNS) metastasis, and poor liver function.

### Sample Size

The sample size was calculated based on a median OS of 8.2 months in the FOLFOX4-placebo group and 14.9 months in the FOLFOX4-ATRA group, as obtained from the data from our retrospective study.^14^ The minimum sample size of each group was 53 patients (two-sided *α* = 0.05; *β* = 0.20; power, 80%; a 10% loss to follow-up was assumed).

### Treatment

For the FOLFOX4-ATRA group, 20 mg ATRA was taken orally 3 times per day for 3 days before FOLFOX4 initiation (oxaliplatin 85 mg/m^2^ iv on Day 1, leucovorin 200 mg/m^2^ iv on Day 1 and Day 2, 5-fluorouracil 400 mg/m^2^ iv bolus at hour 2 and then 600 mg/m^2^ iv over 22 hours on Day 1 and Day 2, once every 2 weeks^9^) and continued for 5 days. For the FOLFOX4-placebo group, placebo (replacing ATRA) was used in the same way as ATRA. Treatment was discontinued if intolerable toxicity or disease progression occurred.

### Efficacy and safety analyses

Liver and renal function tests, routine blood examination, alpha-fetoprotein (AFP) levels, electrocardiogram (ECG), chest X-ray, and abdominal ultrasound were measured at baseline, before each chemotherapy cycle, and then once every 2 ± 1 months during the follow-up phase. Objective responses were assessed at least 2 weeks after the last chemotherapy cycle. For tumor evaluation, CT or MRI was carried out according to the Revised Response Evaluation Criteria in Solid Tumors (mRECIST)^25^ at baseline, 3 ± 1 weeks after the last chemotherapy cycle, and once every 2 ± 1 months during follow-up. If the patients suffered progression, other anticancer treatments were offered based on the patients’ condition. The efficacy was evaluated based on the mRECIST criteria. For patients with both hepatic tumors and extrahepatic lesions, CR was defined when all the intra/extrahepatic lesions had achieved CR, PR was defined when intrahepatic lesions had achieved PR combined with extrahepatic lesions having achieved CR/PR, or extrahepatic lesions having achieved PR combined with intrahepatic lesions having achieved CR/PR, SD was defined as SD of intrahepatic lesions combined with CR/PR/SD of extrahepatic lesions, or SD (stable disease) of extrahepatic lesions combined with CR/PR/SD of intrahepatic lesions; PD (progressive disease) was defined as either extrahepatic lesions or intrahepatic lesions developing PD.

Overall survival (OS) was the primary endpoint, which was defined as the duration between randomization and the time of death from any cause. PFS, ORR, and disease control rate (DCR) are the secondary endpoints.

Study drug toxicities were assessed once every 2 weeks during the treatment phase and were evaluated via vital signs, physical/neurological examination, ECG, Echo, and clinical laboratory tests. All AEs were assessed based on the NCI CTCAE (Version 4.0)^24^ while the subject remained in this study.

### 3D-DIA proteomics assays

Prior to the start of the first cycle of chemotherapy and at the end of treatment, peripheral blood samples of consented patients were taken for 3D-DIA proteomics assays by Jingjie PTM BioLab (Hangzhou, China) Co. Ltd. The standard operational protocols were used for protein extraction, trypsin digestion, HPLC fractionation, and LC‒MS/MS analysis. (Details in supplementary Materials and Methods).

### Proteomics and data analysis

We used the 3D-DIA Proteomics Assays to measure the relative abundance of proteins based on their spectral counts. We excluded proteins that were undetectable in more than 40 samples from the analysis. We normalized the spectral counts by the total number of spectra in each sample and then log-transformed them to obtain the protein expression values.

We performed linear mixed model (LMM) regression analysis using the lme4 R package^26^ to compare the protein expression values across different factors. The factors included response evaluation (PD, SD, PR, or CR), time point (pre- or post-treatment), and treatment group (FOLFOX4-ATRA or FOLFOX4-Placebo). We also included patient ID as a random effect to account for the paired samples from the same patient. For the LMM regression analysis, we used a Gaussian family with an identity link function and specified the fixed and random effects using the formula: protein_expression ~ response_evaluation + treatment_group + time_point (pre- and post-treatment) + (1|patient_ID). We used the glht function from the multcomp R package^27^ to test for the following contrasts: (1) The differences in protein expression between any two response evaluations at the pre-treatment within each treatment arm. (2) The interaction effects of the treatment arm and response evaluation at the pre-treatment, to identify proteins that were uniquely differentially expressed in the FOLFOX4-ATRA arm compared to the FOLFOX4 alone (FOLFOX4-Placebo) arm. (3) The differences in protein expression between pre- and post-treatment within each response evaluation and treatment arm. (4) The interaction effects of the treatment arm and time point within each response evaluation, to identify proteins that were uniquely affected by the FOLFOX4-ATRA treatment compared to the FOLFOX4 alone treatment. *p* values and coefficients were reported from the regression analysis for each contrast. We adjusted the *p* values for multiple testing using the Benjamini-Hochberg method.

For the specific markers for responses to FOLFOX4-ATRA treatment, a protein was considered to be significantly differentially expressed across the different response evaluations if the raw *p* value was <0.05, and then this protein was also considered to be uniquely differentially expressed in the FOLFOX4-ATRA arm if the adjusted *p* value was <0.05 or its absolute value of coefficient >1.5, and its *p* value of the interaction effects of treatment arm and response evaluation at the pre-treatment was less 0.05.

For the potential targets of FOLFOX4-ATRA treatment, a protein was considered to be significantly differentially expressed between pre- and post-treatment within one type of response evaluation and treatment arm if the raw *p* value was <0.05, and then this protein was considered to be uniquely differentially expressed in the FOLFOX4-ATRA arm if the adjusted *p* value was <0.05 or its absolute value of coefficient >1.2, and its *p* value of the interaction effects of treatment arm and time point within the type of response evaluation was less 0.05.

### Statistical analyses

All randomized subjects were included in the intention-to-treat (ITT) analysis of efficacy and patients included in the safety assessment must have received at least one cycle of chemotherapy. For baseline data of the two arms, continuous variables were compared by *t* tests and Mann‒Whitney *U* tests, and categorical variables were compared by the chi-square test. OS and PFS were compared by a stratified log-rank test, and the Kaplan‒Meier method was applied to estimate the OS and PFS curves. The *X*^2^ test or Fisher’s test was applied to compare ORR and DCR between the two groups. A *p* value of <0.05 was considered statistically significant. All analyses were carried out using Stata 12.0 software (StataCorp, Texas 77845 USA).

## Results

### Patient characteristics and treatment

**Fig. 1:**
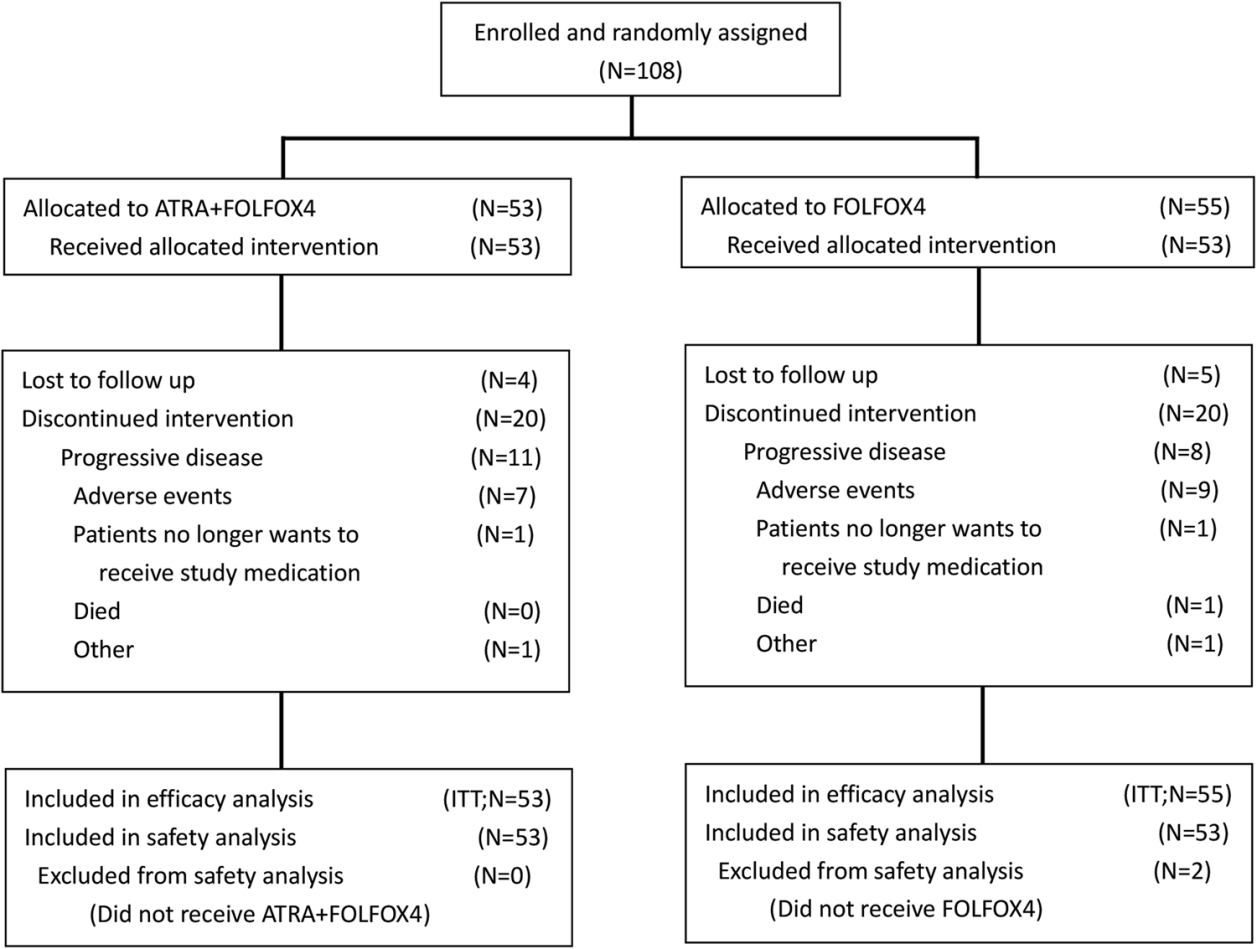
Flow diagram of patient disposition. ATRA all-trans retinoic acid, FOLFOX4 infusional fluorouracil, leucovorin, and oxaliplatin

From October 2017 to September 2021, a total of 116 patients met the inclusion criteria, of which eight patients were excluded. Finally, 108 patients were randomly assigned to the FOLFOX4-ATRA group (*n* = 53) and the FOLFOX4-placebo group (*n* = 55). The included patients were from the Eastern Hepatobiliary Surgery Hospital (92, 85.2%), the Fujian Provincial Cancer Hospital (12, 11.1%), and Zhejiang Sian International Hospital (4, 3.7%) (The ITT population; Fig. 1). Of these, 2 patients decided to withdraw from this study soon after starting the first cycle of FOXFOX4 (the FOLFOX4-placebo group, *n* = 2; the FOLFOX4-ATRA group, *n* = 0). These patients were still included in the ITT analysis, but they were excluded from the safety analysis. There were no significant differences in baseline characteristics between the two treatment arms (Table 1). The most common metastatic sites of HCC were the lung (84, 77.8%), abdominal cavity (14, 13.0%), lymph nodes (10, 9.3%), and bones (7, 6.5%), and there were no significant differences between the two treatment arms (*p* = 0.432). The median number of treatment cycles was 3.9 ± 2.1 for the FOLFOX4-placebo group and 4.0 ± 2.0 for the FOLFOX4-ATRA group (*p* = 0.887). There was no difference between the two treatment arms in the subsequent treatment for patients who were assessed to have PD, and targeted therapy and/or immunotherapy were commonly applied if liver function could be tolerated.

**Table 1:** Baseline patient demographics and clinical characteristics in ITT population (*n* = 108)

Variables	ATRA-FOLFOX (*n* = 53)	FOLFOX (*n* = 55)	*P* value
Age	51.8 ± 10.8	51.8 ± 12.3	0.930
Sex			0.449
Male	47 (88.7%)	46 (83.6%)	
Female	6 (11.3%)	9 (16.4%)	
HBV-DNA			0.447
Negative	17 (32.1%)	14 (25.5%)	
Positive	36 (67.9%)	41 (74.5%)	
α-fetoprotein			0.363
≥ 400 μg/l	22 (41.5%)	26 (47.3%)	
< 400 μg/l	31 (58.5%)	29 (52.7%)	
Platelet			0.333
≥ 100 × 10^9^/L	35 (66.0%)	41 (74.5%)	
< 100 × 10^9^/L	18 (34.0%)	14 (25.5%)	
White blood cell			0.241
≥ 4.0 × 10^9^/L	34 (64.2%)	41 (74.5%)	
< 4.0 × 10^9^/L	19 (35.8%)	14 (25.5%)	
Alanine aminotransferase			0.221
> 44 U/L	5 (9.4%)	2 (3.6%)	
≤ 44 U/L	48 (90.6%)	53 (96.4%)	
Total bilirubin			0.459
> 17.1 μmol/L	13 (24.5%)	17 (30.9%)	
≤ 17.1 μmol/L	40 (75.5%)	38 (69.1%)	
Hepatic tumor stage			0.253
T0/T1	27 (50.9%)	24 (43.6%)	
T2	7 (13.2%)	4 (7.3%)	
T3	3 (5.7%)	9 (16.4%)	
T4	16 (30.2%)	18 (32.7)	
Regional lymph node stage			0.468
N0	47 (88.7%)	51 (92.7%)	
N1	6 (11.3%)	4 (7.3%)	
Metastatic organs			0.432
Lung	41(77.4%)	43(78.2%)	
Bone	3 (5.7%)	4 (7.3%)	
Abdominal	4 (7.5%)	10 (18.2%)	
Other	6 (11.3%)	4 (7.3%)	
Number of extrahepatic metastatic lesions			0.733
≥3	34 (64.1%)	37 (67.3%)	
<3	19 (35.9%)	18 (32.7%)	
Portal vein tumor thrombus (PVTT)			0.776
Yes	16 (25.9%)	18 (15.8%)	
ABLI score			0.339
1	28 (52.8%)	29 (52.7%)	
2	23 (43.4%)	26 (42.3%)	
3	2 (3.8%)	0 (0.0%)	
Chemotherapy cycles	4.0 ± 2.0	3.9 ± 2.1	0.887
Treatments of extrahepatic lesions before enrolled			0.222
None	37 (69.8%)	44 (80.0%)	
Targeted therapy and /or immunotherapy	16 (30.2%)	11 (20.0%)	

### Treatment efficacy

**Fig. 2:**
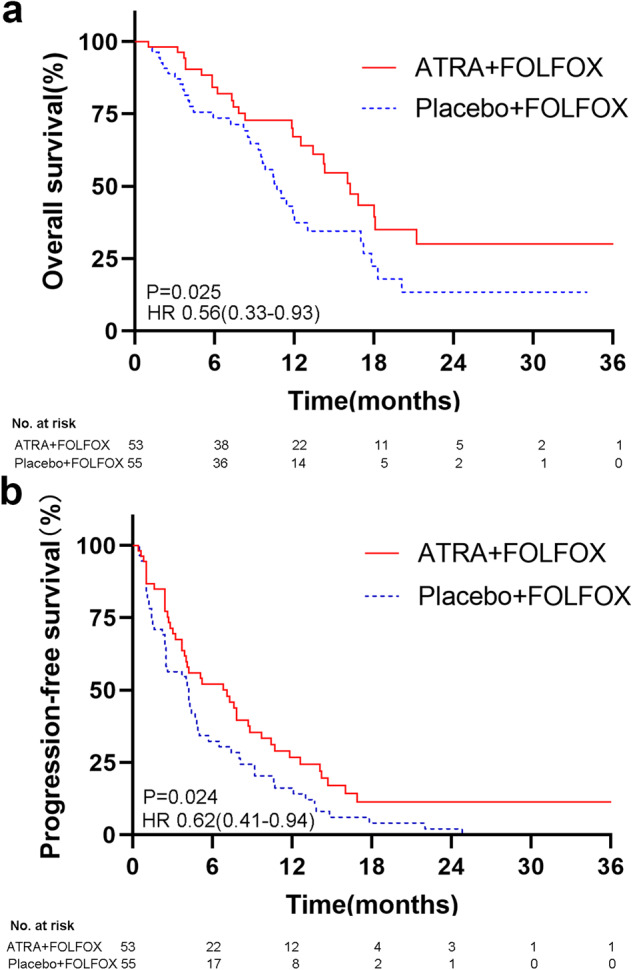
Kaplan–Meier curves showing **a** overall survival and **b** progression-free survival in the intent-to-treat population at final analyses. HR hazard ratio

The median OS was 16.2 months in the FOLFOX4-ATRA group compared with 10.7 months in the FOLFOX4-placeo group (HR 0.56, 95% CI 0.33–0.93; *p* = 0.025) in the ITT population. The corresponding 6-month, 1-year and 2-year survival rates were 73.6%, 41.5%, and 9.4% versus 65.5%, 25.5%, and 3.6%, respectively (Fig. 2a). The multivariate analysis showed that FOLFOX4-placebo (*p* = 0.037, HR 1.740, 95% CI 1.033–2.931) and T stage (*p* = 0.035, HR 1.178, 95% CI 1.012–1.371) were independent prognostic factors of OS (supplementary Table S1).

The median PFS in the ITT population was 7.1 months and 4.2 months for the FOLFOX4-ATRA group and the FOLFOX4-placebo group, respectively (HR 0.62, 95% CI 0.41–0.94; *p* = 0.024). The corresponding 6-month, 1-year, and 2-year PFS rates were 43.4%, 22.5%, and 5.7%, versus 30.9%, 14.5%, and 1.8%, respectively (Fig. 2b). The multivariate analysis showed only FOLFOX4-placebo (*p* = 0.037, HR 1.541, 95% CI 1.026–2.315) to be an independent prognostic factor of PFS (supplementary Table S2).

**Fig. 3:**
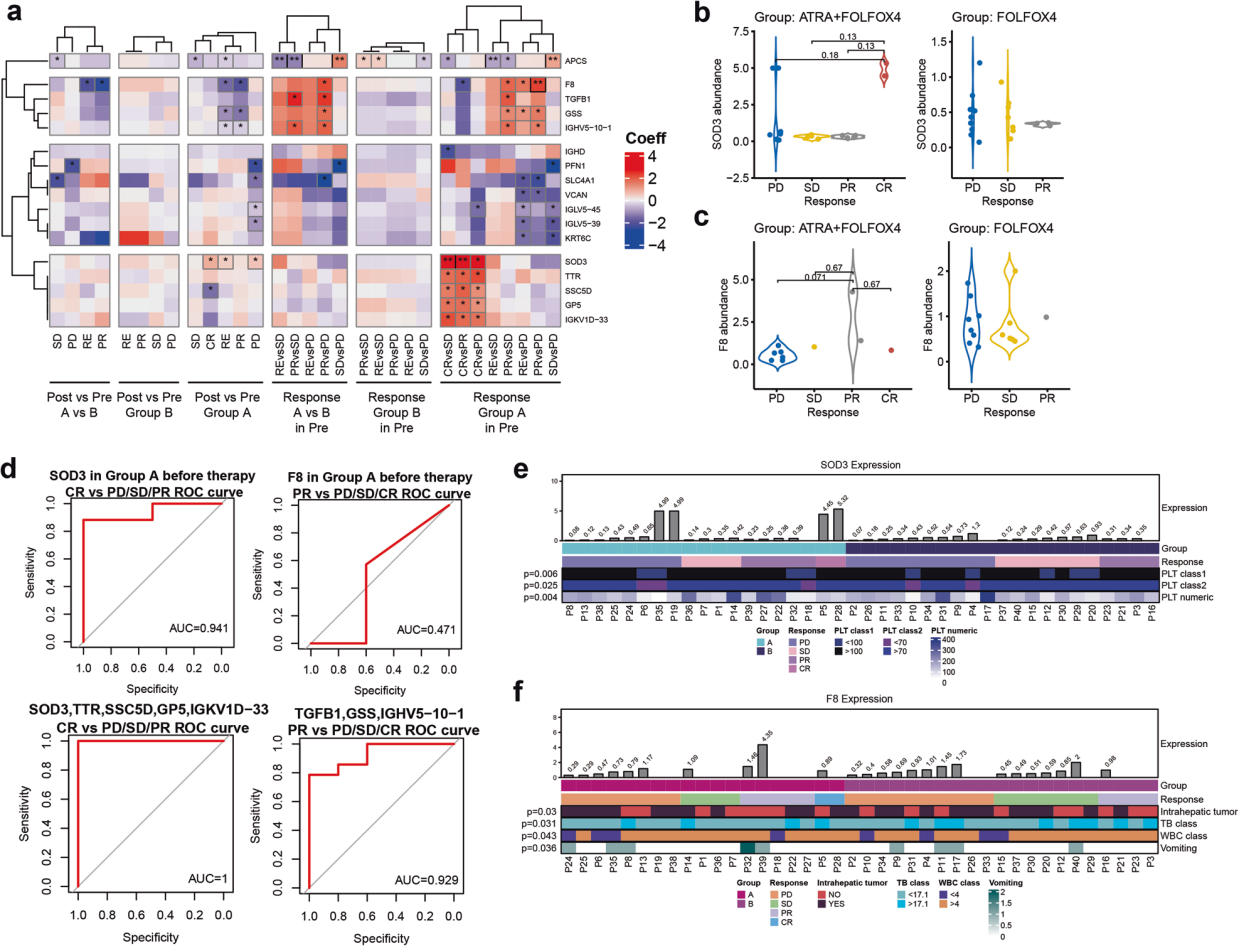
Proteins that differed significantly between FOLFOX4-ATRA responders and non-responders prior to treatment (adjusted *p* < 0.05 or Coefficient absolute value >1.5). **a** Heatmap of significantly distinct proteins illustrating the difference between group A (FOLFOX4-ATRA) and group B (FOLFOX4-placebo) patients for two-way comparisons between PD, SD, PR, CR, and RE (CR + PR), as well as the change in their differential expressions in the pre and post therapy. The color of the heatmap represents the regression coefficient in the linear regression test, with >0 indicating upregulation and less than 0 indicating downregulation. * indicates *p* < 0.05 before multiple testing correction, ** indicates *p* < 0.05 after correction. **b**, **c** Differences in the expression of SOD3 (**b**) and F8 (**c**) in groups A (FOLFOX4-ATRA) and B (FOLFOX4-placebo) of patients with varying responses prior to treatment. Note. The *p* values displayed in Boxplot are calculated using the Wilcox test, which differs from the *p* values calculated using the linear regression test. **d** ROC curves for the prediction of PR or CR patients utilizing SOD3 and F8 and gene panels containing proteins that are uniquely elevated. **e**, **f** The expression of SOD3 (**e**) and F8 (**f**) in each patient prior to therapy was significantly correlated with clinical features (Spearman Correlation test, *p* < 0.05). The top panel is a histogram of *p*rotein expression, while the bottom panel is a heatmap of clinical features

**Fig. 4:**
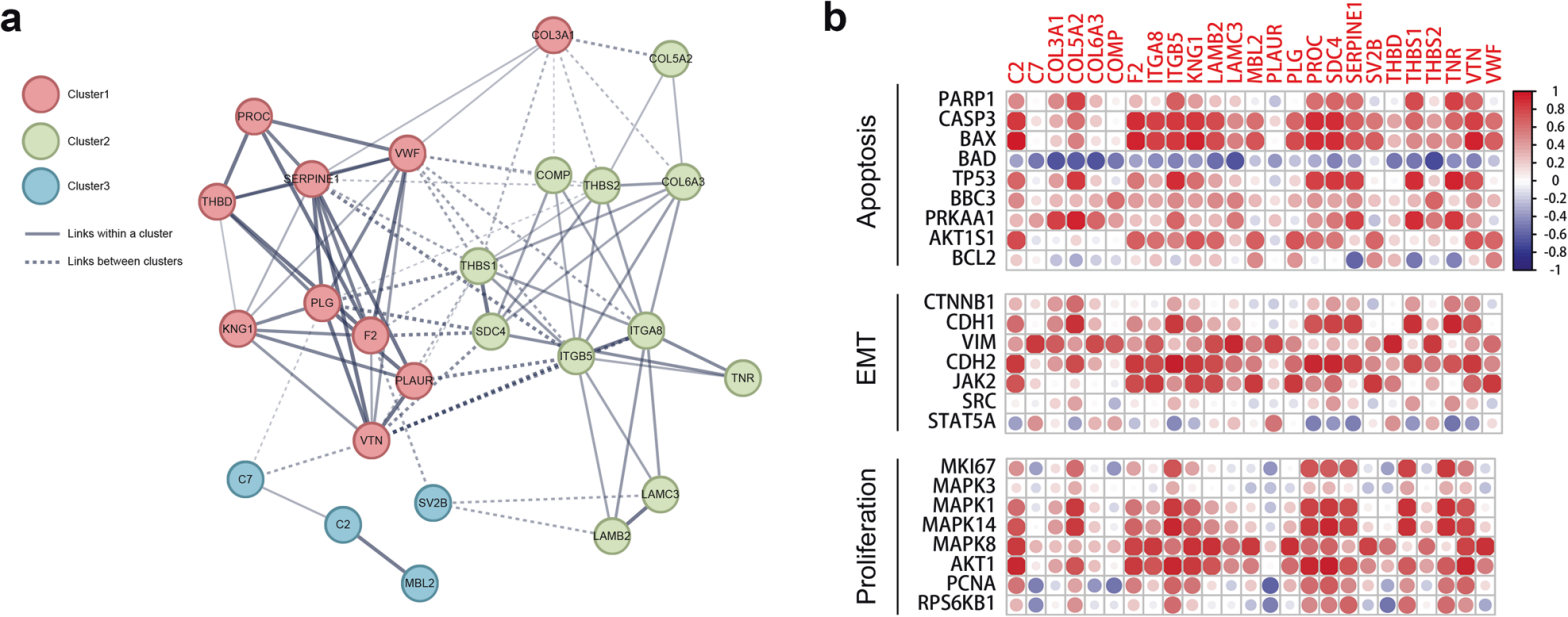
Cluster analysis and correlation analysis of the significantly different expression genes from tumor tissues within ECM receptor interaction and complement & coagulation cascades signalings. **a** The gene-gene interaction network of the treatment response-related genes involved in ECM receptor interaction and complement & coagulation cascades signalings in tumors. The gene connection clusters are colored red (Cluster 1), green (Cluster 2), and blue (Cluster 3). The solid and the dotted lines indicate connection within the same and different clusters respectively. **b** A heatmap showing the correlation of the 25 treatment response-related genes with the oncological features including apoptosis, EMT, and proliferation in tumors (Spearman Correlation test, *p* < 0.05)

The ORR of the FOLFOX4-ATRA group was 24.5%, which included 4 cases of CR, and the DCR was 50.9% (Table 2). The ORR of the FOLFOX4-placebo group was 9.1%, which included only 1 case of CR, and the DCR was 30.9%. The radiological images of two HCC patients with EHM who achieved CR in the FOLFOX4-ATRA group are shown in Figs. 3 and 4. The first patient (supplementary Fig. S1) suffered from lung metastases 2 years after liver tumor resection, and the pulmonary lesions disappeared after 6 cycles of FOLFOX4-ATRA. The second patient (supplementary Fig. S2) suffered from abdominal metastases one and a half years after liver tumor resection, and the abdominal lesions disappeared after 6 cycles of FOLFOX4-ATRA.

**Table 2:** Disease response in ITT population

Parameter	FOLFOX4-ATRA (*n* = 53)		FOLFOX4-placebo (*n* = 55)		P*
	No.	%	No.	%	
^†^ORR	13	24.5	5	9.1	0.031
^‡^DCR	27	50.9	17	30.9	0.034
CR^§^	4	7.5	1	1.8	
PR^§^	9	17.0	4	7.3	
SD^§^	14	26.4	12	21.8	
PD^§^	19	35.8	31	56.4	
Not evaluable	7	13.2	7	12.7	

*ATRA* all-trans-retinoic acid, *CR* complete response, *DCR* disease control rate, *FOLFOX4* infusional fluorouracil, leucovorin, and oxaliplatin, *PD* progressive disease, *ORR* objective response rate

*Cochran–Mantel–Haenszel test. ^†^Defined as complete response (CR) plus partial response (PR). ^‡^Defined as complete response (CR) plus partial response (PR) plus stable disease (SD) ^§^*P* values not determined for individual parameters

### Treatment safety

No significant differences between the two treatments were found for patients who developed AEs and patients who developed AEs of grade 3/4 severity (Table 3). Grade 1/2 headache with an incidence of 20.8% was a unique AE of ATRA, but these headaches were tolerable and did not require any medication, and no patients in the FOLFOX4-ATRA group required dose reduction. The most common AEs in both groups were hematologic and gastrointestinal AEs, which were related to FOLFOX4. Chemotherapy had little impact on liver and kidney functions in the two groups.

**Table 3:** Treatment-emergent AEs in the safety population

Summary of safety events	FOLFOX4-ATRA (*n* = 53)					FOLFOX4-placebo (*n* = 53)				
	No.		%			No.			%	*p*
Any AE	48		90.6			48			90.6	1.000
AE grade ≥ 3	21		39.6			25			47.2	0.433
Discontinuation	7		13.2			9			17.0	0.587

Individual AEs	All AEs					Grade 3–4 AEs				
	ATRA-FOLFOX4 (*n* = 53)			FOLFOX4-placebo (*n* = 53)		ATRA-FOLFOX4 (*n* = 53)		FOLFOX4-placebo (*n* = 53)		
	No.	%		No.	%	No.	%	No.		%
Hematologic										
Neutropenia	37	69.8		34	64.2	18	34.0	18		34.0
Leukocytopenia	39	73.6		32	60.4	13	24.5	11		20.8
Thrombocytopenia	22	41.5		21	39.6	4	7.5	6		11.3
Anemia	10	18.9		12	22.6	1	1.9	2		3.8
Non-hematologic										
Nausea	18	34.0		27	50.9	3	5.7	4		7.5
Vomiting	9	17.0		13	24.5	1	1.9	1		1.9
Headache	11	20.8		3	5.7	0	0.0	0		0.0
AST	6	11.3		6	11.3	0	0.0	2		3.8
Bilirubin	0	0.0		2	3.8	0	0.0	0		0.0
Diarrhea	7	13.2		7	13.2	2	3.9	0		0.0
ALT	6	11.3		5	9.4	0	0.0	2		3.8
Sensory neuropathy	3	5.7		9	17.0	0	0.0	2		3.8
Allergy	1	1.9		0	0.0	0	0.0	0		0.0
Fever	1	1.9		1	1.9	0	0.0	0		0.0
Creatinine	0	0.0		0	0.00	0	0.0	0		0.0

*AE* adverse event *ATRA* all-trans retinoic acid, *FOLFOX4* infusional fluorouracil, leucovorin, and oxaliplatin, *ALT* alanine aminotransferase, AST aspartate transaminase

### Specific markers for response to FOLFOX4-ATRA therapy

We performed an exploratory study to identify molecular markers that could predict the efficacy of FOLFOX4-ATRA treatment at the pre-treatment. We measured the protein expression in blood samples from patients with different response evaluations (PD, SD, PR, and CR) on the two treatment arms using the 3D-DIA proteomics assays. We compared the protein expression between any two response evaluations within each treatment arm and identified 80 proteins that were significantly differentially expressed across the different response evaluations (*p* value [raw] <0.05 and no limit in coefficient, supplementary Fig. S3). The proteins related to responses to FOLFOX4-ATRA treatment and that were significantly upregulated in CR, PR, PD, and SD patients were enriched in ECM receptor interaction, complement and coagulation cascades, antigen binding, and endopeptidase regulator activity, respectively, according to the Gene Ontology and KEGG pathway enrichment analysis (supplementary Fig. S3).

Then we further analyzed the interaction effects of treatment arm and response evaluation at the pre-treatment and identified 17 proteins that were uniquely differentially expressed in the FOLFOX4-ATRA arm compared to the FOLFOX4 alone arm (adjusted *p* < 0.05 or absolute value of coefficient >1.5, Fig. 3a). Among those 17 proteins, superoxide dismutase 3 (SOD3) was specifically upregulated in CR patients (Fig. 3b), while coagulation factor VIII (F8) was specifically upregulated in PR patients in the FOLFOX4-ATRA arm (Fig. 3c). SOD3 belongs to the superoxide dismutase (SOD) protein family and is an extracellular antioxidant defense against oxidative damage. F8 participates in the intrinsic pathway of blood coagulation and functions as a cofactor for factor IXa, which converts Factor X to the activated form Xa. We performed ROC curve analysis and found that the expression of SOD3 was a good predictor of CR in patients (AUC = 0.941), but F8 was not a good predictor of PR (AUC = 0.471), as it was not detected in some samples when the deletion was assigned a value of 0. We also constructed a panel of multiple proteins that were specifically upregulated in CR patients (SOD3, TTR, SSC5D, GP5, IGKV1D-33) or in PR patients (TGFB1, GSS, IGHV5-10-1) in the FOLFOX4-ATRA arm and found that they effectively predicted the responses to the FOLFOX4-ATRA treatment (Fig. 3d). Moreover, we observed that SOD3 and F8 expressions were correlated with several clinical parameters, such as PLT, TB, and WBC (Fig. 3e, f), indicating that clinical characteristics can be exploited to facilitate the use of these proteins as predictors. These results suggest that these proteins are important determinants of response to the FOLFOX4-ATRA treatment.

In addition, we also compared the differences in gene expression between the tumors of the responsive (CR, PR) and nonresponsive (PD) patients by transcriptome sequencing. There were 1017 genes significantly upregulated and 916 genes significantly downregulated in the response group vs. the nonresponse group. By analyzing the same signaling pathways of enriched genes from tumor tissues and proteins from plasma, we found that the significant genes and proteins regulated by FOLFOX4-ATRA were involved in ECM receptor interaction and complement and coagulation cascade signaling according to the Gene Ontology and KEGG pathway enrichment analysis. Furthermore, the differentially expressed genes from tumor tissues within these two signaling pathways can be grouped into three clusters according to the STRING database (Fig. 4a). These genes were significantly correlated with the expression of the star genes that were involved in apoptosis, epithelial-mesenchymal transition, and proliferation (Fig. 4b).

### Potential targets for FOLFOX4-ATRA therapy

We performed a differential expression analysis to identify proteins that were affected by the FOLFOX4-ATRA treatment. We compared the protein expression between pre- and post-treatment within each response evaluation and treatment arm and identified 184 proteins that were significantly differentially expressed after the FOLFOX4-ATRA treatment (*p* value [raw] <0.05 and no limit in coefficient, supplementary Fig. S4). PD, SD, PR, and CR patients showed distinct protein expression patterns after FOLFOX4-ATRA treatment. Proteins that were significantly downregulated in CR, PR, and SD patients were enriched in complement and coagulation cascades according to the Gene Ontology and KEGG pathway enrichment analyses (supplementary Fig. S4).

**Fig. 5:**
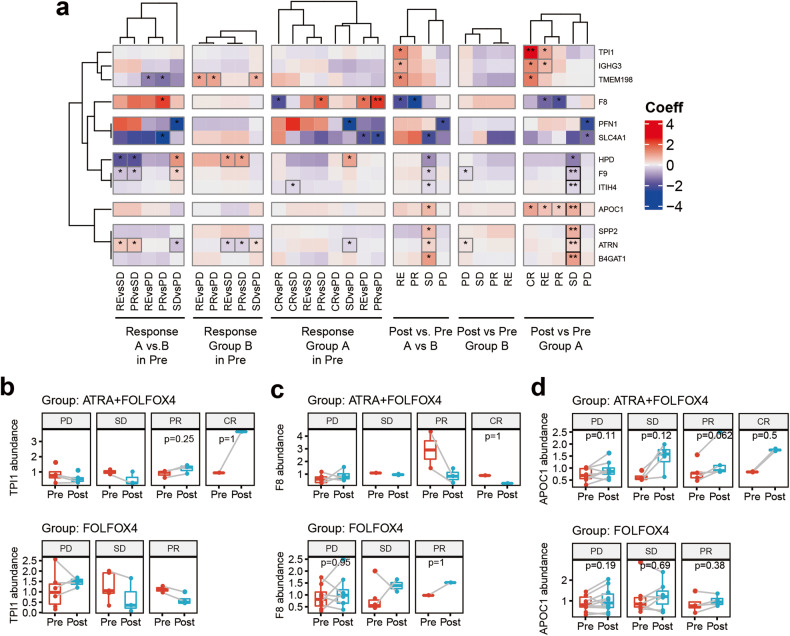
Proteins that differed significantly between the pre and post FOLFOX4-ATRA treatment (adjusted *p* < 0.05 or coefficient absolute value > 1.2). **a** Heatmap of significantly distinct proteins illustrating the change in their differential expression in the pre and post therapy, as well as the difference between group A (FOLFOX4-ATRA) and group B (FOLFOX4-placebo) patients for two-way comparisons between PD, SD, PR, CR, and RE (CR + PR). The color of the heatmap represents the regression coefficient in the linear regression test, with >0 indicating upregulation and <0 indicating downregulation. * indicates *p* < 0.05 before multiple testing correction, ** indicates *p* < 0.05 after correction. **b**–**d** TPI1 (**b**), F8 (**c**) and APOC1 (**d**) expression levels in PD, SD, PR, and CR patients pre and post therapy with FOLFOX4-ATRA and treatment with FOLFOX4-placebo. Note. The *p* values displayed in Boxplot are calculated using the Wilcox test, which differs from the *p* values calculated using the linear regression test

Further, we analyzed the interaction effects of treatment group and time point within each response evaluation and identified 13 proteins that were uniquely differentially expressed in the FOLFOX4-ATRA group compared to the FOLFOX4 alone group (adjusted *p* < 0.05 or absolute coefficient value >1.2, Fig. 5a). Among those 13 proteins, triosephosphate isomerase 1 (TPI1) was specifically upregulated in CR patients, and coagulation Factor F8 was specifically downregulated in PR patients after FOLFOX4-ATRA treatment (Fig. 5b, c). TPI1 can catalyze the isomerization of glyceraldehyde 3-phosphate (G3P) and dihydroxy-acetone phosphate (DHAP) in glycolysis and gluconeogenesis. Notably, coagulation Factor F8 was previously identified to be specifically elevated in PR patients before the treatment in the FOLFOX4-ATRA group. Moreover, apolipoprotein C1 (APOC1), which is related to plasma lipoprotein assembly, remodeling, metabolism, and NR1H2- and NR1H3-mediated signaling, was significantly increased in SD, PR, and CR patients after FOLFOX4-ATRA treatment.

## Discussion

To our knowledge, this study represents the first published clinical trial on the treatment of HCC patients with EHM using a combination therapy of FOLFOX4 and ATRA. The treatment showed significant clinical benefits to this group of HCC patients compared with FOLFOX4-placebo. This study also verified the results of our previously published retrospective study. The median OS was 16.2 months in the FOLFOX4-ATRA group, which was significantly higher than that in the FOLFOX4-placebo group. Furthermore, the secondary endpoints, including a median PFS of 7.2 months, ORR of 24.5%, and DCR of 50.9%, were also very encouraging. The toxicity in this study was consistent with our previous experience with FOLFOX4 in treating HCC patients. The results of this study provide a safe and effective treatment for HCC patients with EHM.

In recent years, immunotherapy for advanced HCC has made great progress. In addition to the IMbrave150 study and HIMALAYA trial, the ORIENT-32 study (sintilimab plus a bevacizumab biosimilar as first-line therapy) and the Keynote-394 study (pembrolizumab as second-line therapy) both disclosed encouraging results in 2021 and 2022.^28^ However, these studies did not disclose the results when we started our study in 2017. In our study, 30.2% of patients in the FOLFOX4-ATRA group had received targeted therapy and/or immunotherapy previously, but FOLFOX4-ATRA still showed encouraging results for these patients. FOLFOX4-ATRA provided a clinical benefit in all planned subgroup analyses, including pre-treatment conditions, age, AFP, T stage, and number of EHMs. The ORR and DCR of these patients were 18.8% and 43.8%, respectively, which were significantly higher than those obtained in the FOLFOX4-placebo group (0% and 18.2%). The results showed that FOLFOX4-ATRA is still applicable to patients who failed first-line treatment. However, further confirmation studies are needed in the future.

BCLC stage C includes HCC patients with EHM and/or portal vein tumor thrombus (PVTT). The staging and treatment of PVTT have made steady progress.^16,18^ However, there are still few studies on EHM. The current research trends on the use of chemotherapy sensitizers and targeted therapy combined with immune therapy are the research directions of EHM treatment in the future.

ATRA is an antiproliferative and cytodifferentiating agent rather than a cytotoxic agent. ATRA was first used in the treatment of hematological tumors, and it has become a standard treatment for APL, resulting in high CR rates of over 90%.^19^ In recent years, ATRA has also made positive progress in the treatment of solid tumors. The mechanisms are more complex and are still under investigation, although many possible genes or pathways have been reported.^20–23^ This multicenter study showed that FOLFOX4-ATRA was well tolerated. ATRA, when administered at a dosage of 25–45 mg/m² per day, has widely been used to treat APL, with headache being the most common side effect. These manifestations were mild in our study and did not require any additional treatment. The grade 3/4 AEs were similar between the two treatment groups in this study, although 13.2% of patients in the FOLFOX4-ATRA group and 16.4% of patients in the FOLFOX4-placebo group discontinued chemotherapy because of AEs of FOLFOX4.

Differential proteins in patients with various responses prior to treatment in the FOLFOX4-ATRA group were analyzed to identify protein indicators that could predict patient prognosis prior to treatment. Using the pre-treatment proteome data in plasma, markers in CR, PR, and SD patients were identified, which showed significant and specifically high expression in each group prior to treatment. The results suggested that when future studies employ these markers in predicting prognosis (CR, PR, or SD), different combinations of these proteins should be considered (SOD3, TTR, SSC5D, GP5, IGKV1D-33 combination for CR and TGFB1, GSS, IGHV5-10-1 combination for PR). In addition, functional enrichment analysis of these markers revealed that patients with CR, PR, and SD have unique, highly expressed proteins in different biological pathways. Complement- and coagulation-related proteins were highly expressed in all these pathways, although different proteins were involved. Among these, alpha 2-antiplasmin (SERPINF2) and Ficolin 3 (FCN3) were significantly expressed in CR, F8, and C4A in PR, and Factor XI (F11) and complement Factor H-related protein 2 (CFHR2) in SD. In vivo and in vitro trials conducted as early as the 1990s have shown ATRA corrects the activities of pro-coagulant and fibrinolytic agents.^29^ ATRA has been utilized frequently in the treatment of APL, and studies have demonstrated the association between ATRA and coagulation. For example, coagulation abnormalities in high-risk patients with APL treated with ATRA were shown to be amenable to treatment via suppression of intrinsic mechanisms that accelerated extracellular chromatin degradation.^30^ Hamed et al.^31^ reported that ATRA, by upregulating thrombomodulin (TM) expression on the surfaces of endothelial cells and causing oscillatory elevation of TM expression on endothelial cells during ATRA treatment, played a role in the treatment of coagulation disorders in cancer patients. All these studies demonstrated that ATRA targeted and affected the coagulation cascade reaction. When differential proteins before and after therapy were compared to screen for any potential targets of FOLFOX4-ATRA treatment in our study, coagulation Factors F8, F9, F12, and complement C6 and C8A were downregulated in CR, PR, and SD patients after treatment. These results suggested that coagulation factors and complement are targets of FOLFOX4-ATRA. Thus, our postulation is that patients with high expression of coagulation-related proteins are more responsive to FOLFOX4-ATRA treatment, as they are more susceptible to ATRA, resulting in a better prognosis.

Several studies^32,33^ have been reported to compare the cost performance of FOLFOX4 and targeted therapy. The results indicated that FOLFOX4 was better than targeted therapy in providing higher effectiveness with significantly lower costs in treating advanced HCC patients. As ATRA used in our study is inexpensive (approximately $45 per cycle), FOLFOX4-ATRA can be accepted in developing countries and areas.

Our study has limitations. First, as headache is a peculiar side effect of ATRA, it could affect the effect of double blindness clinically. The medical care and follow-up may have differed for patients who developed headaches, which could have affected the results. However, the incidence of headaches was only ~20%, and all data in the study were collected and analyzed by masked experts to minimize biases. Second, concomitant therapies were inevitable, especially for patients who were assessed to have PD. Although different treatments were given according to different conditions, there were no significant differences in postchemotherapeutic treatments given to these two groups of patients. Third, our study included both first-line and second-line patients, which would make the enrolled population heterogeneous, but the subgroup analysis revealed that ATRA-FOLFOX is efficient both for first-line and second-line patients. Last, due to the differences in the actual patient enrollment rates among the different centers, there are indeed differences in the number of patients enrolled in each center, which could affect the results slightly.

## Conclusion

In eastern China, FOLFOX4-ATRA achieved better survival outcomes than FOLFOX4-placebo in treating advanced HCC patients with EHM without any additional severe AEs.

### Supplementary information

Supplementary Materials

CONSORT Checklist

## Data Availability

The data that support the findings of this study are available from the corresponding author upon reasonable request.
