# Supplementary material for: Combined FOLFOX4 with all-trans retinoic acid versus FOLFOX4 with placebo in treatment of advanced hepatocellular carcinoma with extrahepatic metastasis: a randomized, double-blind comparative study
Source: Signal Transduct Target Ther. 2023 Sep 27;8:368. doi: 10.1038/s41392-023-01604-3 (PMC10522582; doi:10.1038/s41392-023-01604-3)
Supplement: Supplementary file 1 — Supplementary Materials [file 41392_2023_1604_MOESM1_ESM.docx]

Supplementary Materials for

Combined FOLFOX4 with all-trans retinoic acid versus FOLFOX4 with placebo in treatment of advanced hepatocellular carcinoma with extrahepatic metastasis: A randomized, double-blind comparative study

Juxian Sun, Feifei Mao, Chang Liu, Fan Zhang, Dafeng Jiang, Weixing Guo, Lei Huo, Liping Zhou, Lau Wan Yee, Jie Shi, Shuqun Cheng

Correspondence to: chengshuqun@aliyun.com

**This PDF file includes:**

Materials and Methods

Figure S1

Figure S2

Figure S3

Figure S4

Table S1

Table S2

**Other Supplementary Materials for this manuscript include the following:**

Final protocol

SAP

Materials and Methods

Collection and Storage of Samples for Proteomics Assays:

Prior to the start of the first cycle of chemotherapy and at the end of treatment, peripheral blood samples of consented patients were taken for proteomics assays. Standard operational protocols were followed when collecting plasma samples. In a nutshell, peripheral blood samples (2 mL per person) were collected in an EDTA anticoagulant tube and centrifuged for 15 minutes at 1,000 g/min. The Plasma was immediately transferred to a clean polypropylene test tube and stored at -80°C after centrifugation.

Extraction, Digestion, and Fractionation of Protein

The cellular debris from the plasma sample was removed by centrifugation at 12,000 g for 10 minutes at 4 °C. The supernatant was then transferred to a fresh centrifuge tube. PierceTM Top 14 Abundant Protein Depletion Spin Columns Kit was used to remove the top 14 high abundance proteins (ThermoFisher Scientific). The protein concentration was measured using a BCA kit as directed by the manufacturer.

The protein solution was reduced with 5 mM dithiothreitol for 30 minutes at 56 °C before being alkylated with 11 mM iodoacetamide for 15 minutes at room temperature in the dark. After that, the protein sample was diluted by adding 100 mM TEAB to a urea concentration of less than 2 M. Finally, trypsin was added at a trypsin-to-protein mass ratio of 1:50 for the first overnight digestion and a trypsin-to-protein mass ratio of 1:100 for the second 4 h digestion. The peptides were then desalted using a C18 SPE column.

Using an Agilent 300 Extend C18 column (5 m particles, 4.6 mm ID, 250 mm length), the sample was separated into fractions by high pH reverse-phase HPLC. The wavelength was 214 nm, and the temperature in the column oven was 35°C. After 30 minutes of washing with 95 percent equilibration buffer A, the peptide sample was fed into HPLC after the baseline was flat. Peptides were separated using a gradient of 2% to 60% acetonitrile in 10 mM ammonium bicarbonate pH 10 and collected at 1 minute in each tube. The 11th to 46th tubes were collected, and 36 tubes were mixed into 9 fractions and vacuum centrifuged to dry.

LC-MS/MS Analysis

The peptides were dissolved in liquid chromatography's mobile phase A and separated using the EASY-nLC 1200 ultra-high performance liquid phase system (ThermoFisher Scientific). Mobile phase A was an aqueous solution with 0.1 percent formic acid and 2% acetonitrile, while mobile phase B was an aqueous solution with 0.1 percent formic acid and 90% acetonitrile. The setting for liquid phase gradient: 0-114 minutes, 4 percent 33 percent phase B; 114-117 minutes, 33 percent 80 percent phase B; 117-120 minutes, 80 percent phase B. The flow rate was kept constant at 500 nL/min. The peptides were separated using an ultra-high performance liquid system, then injected into an NSI ion source for ionization before being evaluated in Q ExactiveTM HF-X (ThermoFisher Scientific).

The liquid phase parameters remained unchanged from when the library was created. Peptides were isolated using an ultrahigh performance liquid chromatography system and evaluated using Q ExactiveTM HF-X mass spectrometry. The high-resolution Orbitrap was used to detect and analyze peptide precursor ions and their secondary fragments. The primary mass spectrometer's scanning range was set at 385-1200 m/z, and the scanning resolution was set to 120000; the secondary scanning resolution was set to 15000. The data acquisition mode employed the data-independent scanning (DIA) program, in which peptide ions were fragmented in the HCD collision cell utilizing 27 percent of the fragmentation energy, followed by secondary mass spectrometry analysis. The secondary spectrum's automatic gain control (AGC) was set to 5E5, and the fixed initial mass was set to 200 m/z.

Protein Annotation in LC-MS/MS

MaxQuant search engine (v.1.6.15.0) was used to process the generated MS/MS data. Tandem mass spectra were compared to the human SwissProt database (20422 entries), which was concatenated with the reverse decoy database. Trypsin/P was chosen as the cleavage enzyme, with up to two missed cleavages permitted. In the first search, the mass tolerance for precursor ions was set to 20 ppm and 5 ppm in the main search, while the mass tolerance for fragment ions was set to 0.02 Da. FDR was adjusted to < 1%.

Proteomics Assays Data Analysis

Data on protein expressions came from two distinct groups (Group A: The FOLFOX4-ATRA group and Group B: the FOLFOX4-placebo group). Samples from the baseline (before treatment) and time point (after treatment) measurements formed 4 experimental subgroups (Subgroup A – before, Subgroup A – after, Subgroup B – before, Subgroup B – after). Samples from patients with PD, SD, PR, and CR were measured for each subgroup.

All protein expression values were normalized by calculating the relative abundance of each protein in each sample. Analyses of differential protein expression were conducted by fitting linear mixed models (LMM) with the lme4 R package25 and proteins whose expression levels were undetectable in more than 40 samples were not analysed The protein expression was a response variable (y), while the explanatory variables were treatment response (PD, SD, PR, and CR), time point (pre and post treatment), and treatment group (Group A and Group B) used as fixed effects. The patient ID was used as a random effect to allow us to account for the samples being paired (same samples measured in the pre and post therapy). To test for differences between different treatment responders to FOLFOX4-ATRA therapy, the generalized linear hypothesis (glht) function from the multcomp R package26 was utilized to test for the following three contrasts: (1) differences in protein expressions between patients with PD, SD, PR, and CR before therapy in Group A, (2) similar differences in Group B, and (3) any interaction when comparing differences between Group A and Group B. To test for the differences in the pre and post FOLFOX4-ATRA therapy, the following three contrasts were absotested: (1) differences in protein expressions in pre and post therapy in Group A, (2) differences in pre and post therapy in Group B, and (3) any interaction when comparing the differences between Groups A and B. The resulting p-values were adjusted using the FDR method to account for multiple comparisons.

Figure. S1.


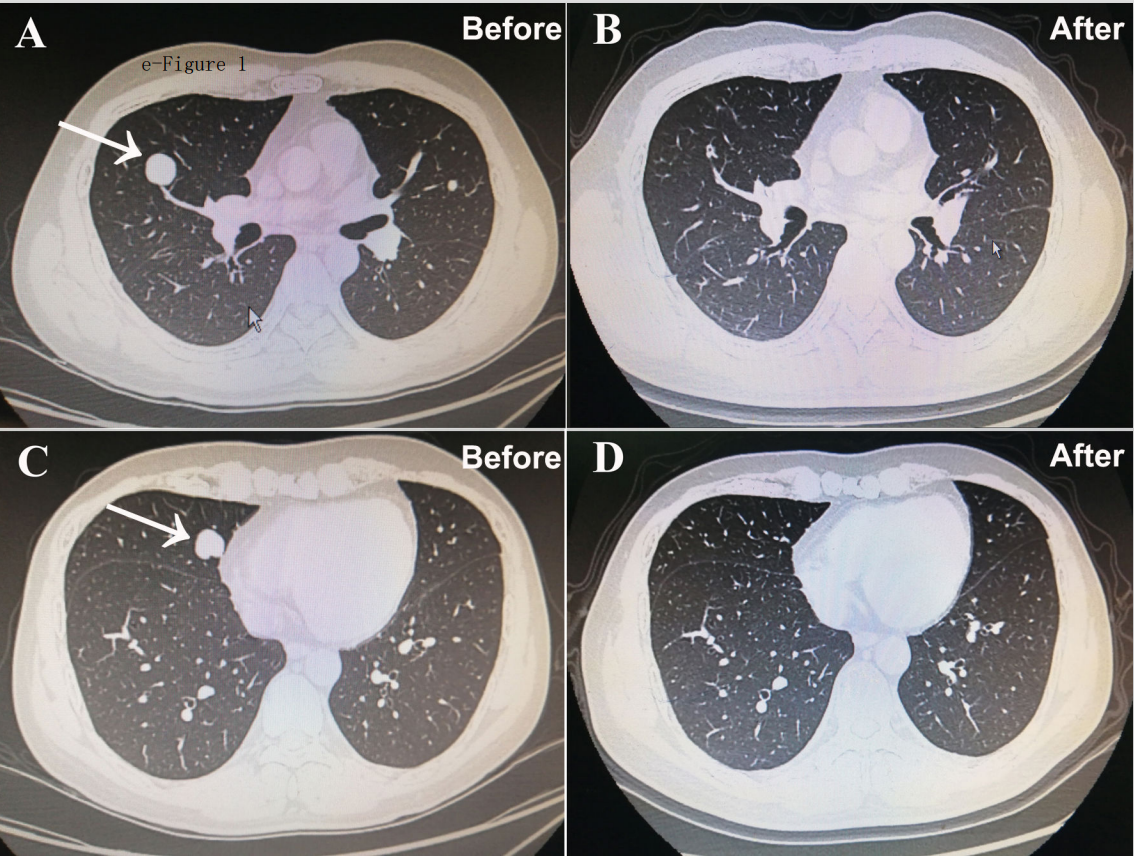


Typical patient 1: A, C: Pulmonary metastasis before treatment; B, D: The lesions disappeared after FOLFOX4-ATRA

Figure. S2.


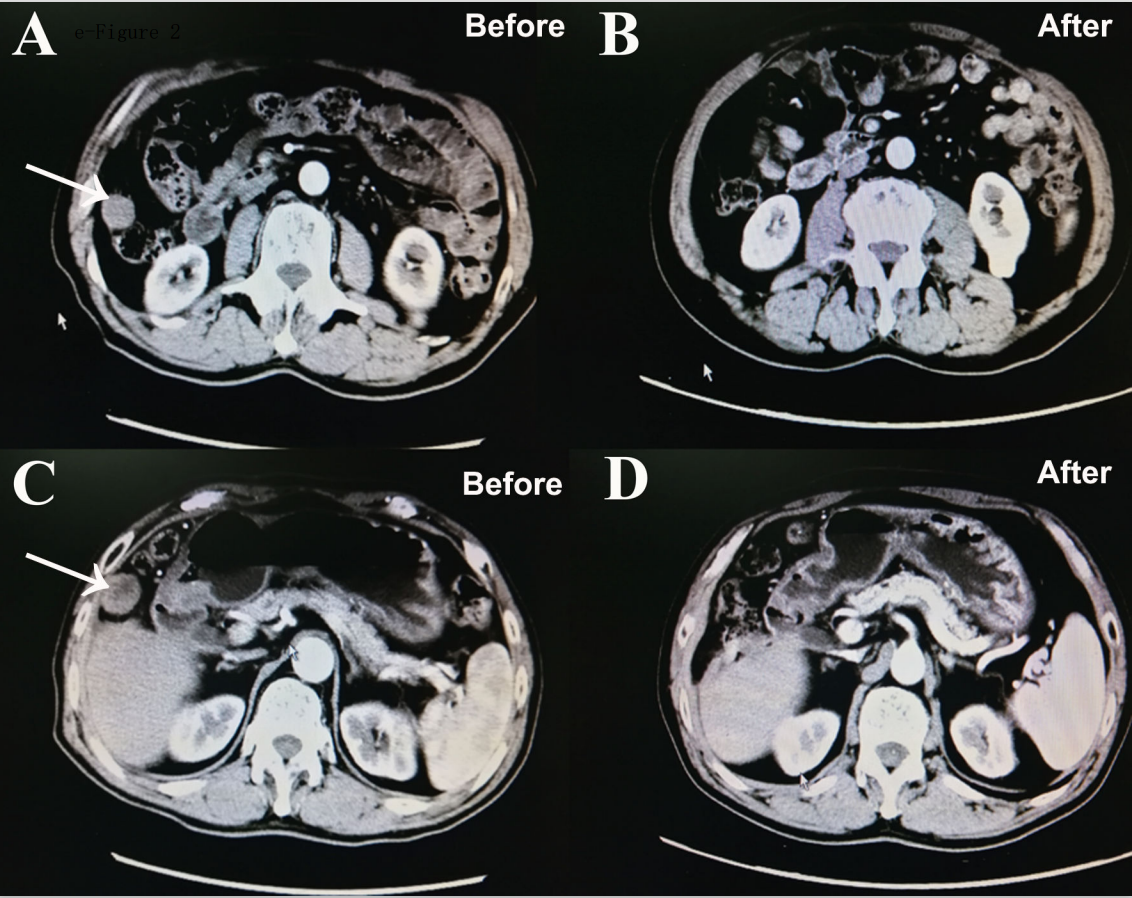


Typical patient 1: A, C: Abdominal metastasis before treatment; B, D: The lesions disappeared after FOLFOX4-ATRA.

Figure. S3.


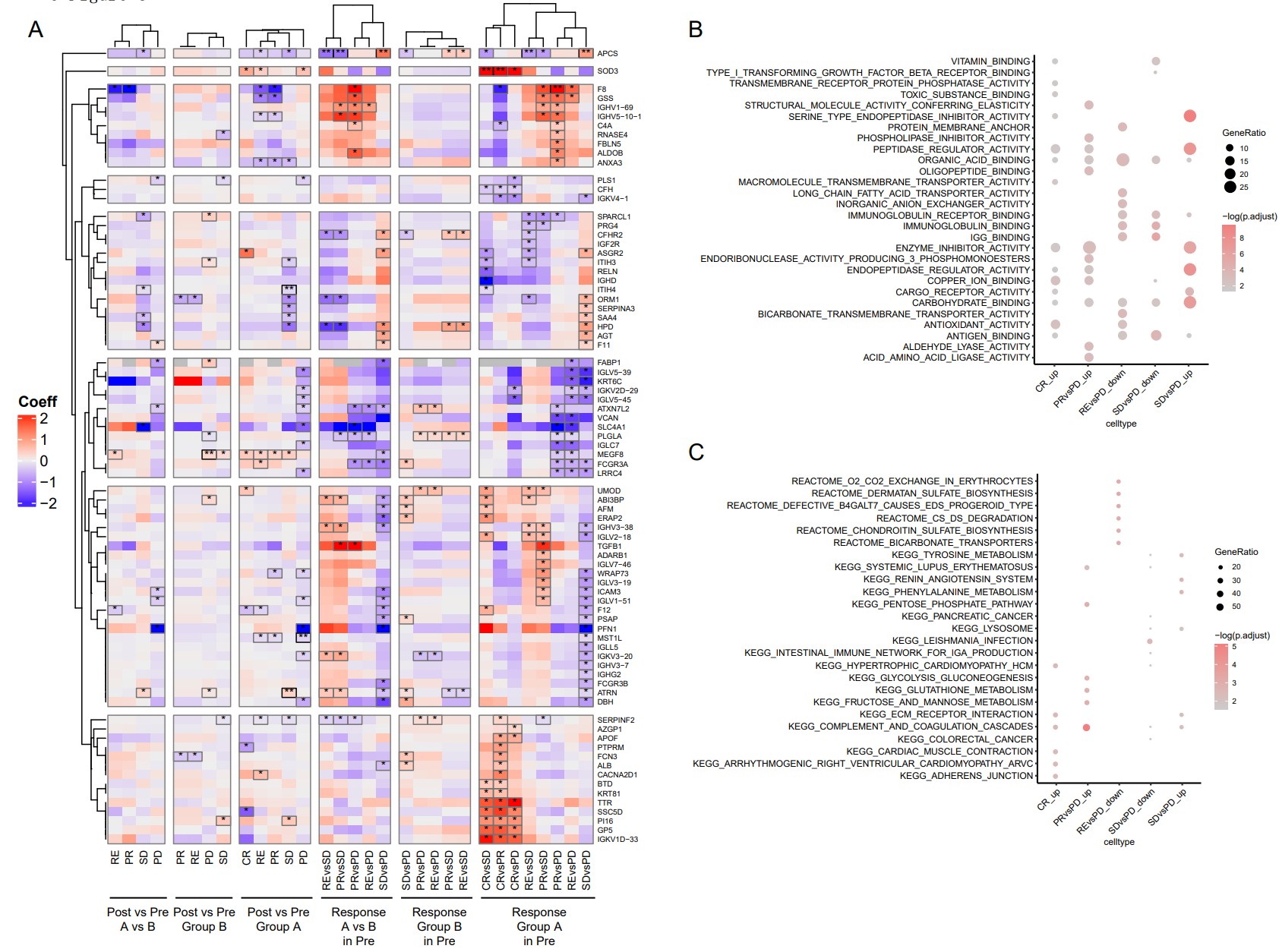


Proteins that differed significantly between FOLFOX4-ATRA responders and non-responders prior to treatment (P value < 0.05). **A,** Heat map of significantly distinct proteins illustrating the difference between group A and group B patients for two-way comparisons between PD, SD, PR, CR, and RE (CR+PR), as well as the change in their differential expression in the pre and post therapy. The color of the heat map represents the regression coefficient in the linear regression test. **B,** the enrichment of Gene Ontology for different protein modules. **C,** The enrichment of KEGG pathways for different protein modules.

**CR_up:** SOD3, SERPINF2, AZGP1, APOF, PTPRM, FCN3, ALB, CACNA2D1, BTD, KRT81, TTR, SSC5D, PI16, GP5, IGKV1D−33. **RE_down**: FABP1, IGLV5−39, KRT6C, IGKV2D−29, IGLV5−45, ATXN7L2, VCAN, SLC4A1, PLGLA, IGLC7, MEGF8, FCGR3A, LRRC4. **PR_up**: F8, GSS, IGHV1−69, IGHV5−10−1, C4A, RNASE4, FBLN5, ALDOB, ANXA3. **SD_down**: UMOD, ABI3BP, AFM, ERAP2, IGHV3−38, IGLV2−18, TGFB1, ADARB1, IGLV7−46, WRAP73, IGLV3−19, ICAM3, IGLV1−51, F12, PSAP, PFN1, MST1L, IGLL5, IGKV3−20, IGHV3−7, IGHG2, FCGR3B, ATRN, DBH. **SD_up**: APCS, SPARCL1, PRG4, CFHR2, IGF2R, ASGR2, ITIH3, RELN, IGHD, ITIH4, ORM1, SERPINA3, SAA4, HPD, AGT, F11.

Figure. S4.


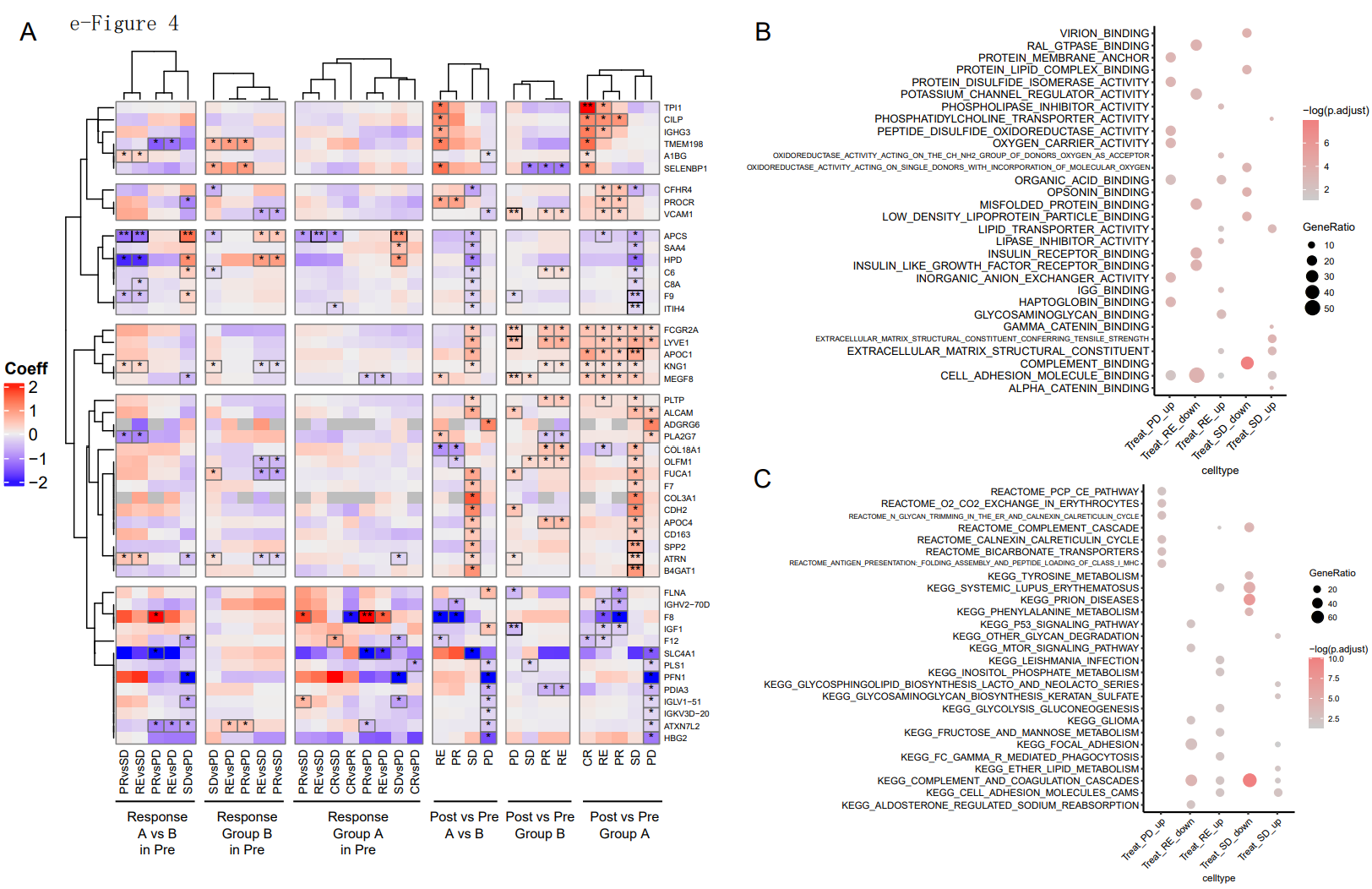


Proteins that differed significantly between pre and post FOLFOX4-ATRA treatment (P value < 0.05). A, Heat map of significantly distinct proteins illustrating the difference between group A and group B patients for two-way comparisons between PD, SD, PR, CR, and RE (CR+PR), as well as the change in their differential expression in the pre and post therapy. The color of the heat map represents the regression coefficient in the linear regression test. B, the enrichment of Gene Ontology for different protein modules. C, The enrichment of KEGG pathways for different protein modules.

**Treat_RE_up:** TPI1, CILP, IGHG3, TMEM198, A1BG, SELENBP1, CFHR4, PROCR, VCAM1, FCGR2A, LYVE1, APOC1, KNG1, MEGF8. **Treat_RE_down:** FLNA, IGHV2−70D, F8, IGF1, F12. **Treat_SD_up:** PLTP, ALCAM, ADGRG6, PLA2G7, COL18A1, OLFM1, FUCA1, F7, COL3A1, CDH2, APOC4, CD163, SPP2, ATRN, B4GAT1. **Treat_SD_down:** APCS, SAA4, HPD, C6, C8A, F9, ITIH4. **Treat_PD_up:** SLC4A1, PLS1, PFN1, PDIA3, IGLV1−51, IGKV3D−20, ATXN7L2, HBG2.

Table S1.

Univariate and Multivariable Analysis of OS

| Variables |  | | Univariate Analysis | |  | | Multivariable Analysis | |
| --- | --- | --- | --- | --- | --- | --- | --- | --- |
|  | p | HR | 95%CI | | p | HR | 95%CI | |
| Folfox-placebo | 0.027 | 1.792 | 1.068 | 3.005 | 0.037 | 1.740 | 1.033 | 2.931 |
| Sex | 0.033 | 2.005 | 1.056 | 3.807 | 0.158 | 1.612 | 0.830 | 3.129 |
| Age | 0.060 | 1.644 | 0.980 | 2.758 |  |  |  |  |
| PVTT | 0.191 | 1.429 | 0.836 | 2.440 |  |  |  |  |
| T stage | 0.013 | 1.207 | 1.040 | 1.401 | 0.035 | 1.178 | 1.012 | 1.371 |
| N stage | 0.964 | 0.979 | 0.391 | 2.451 |  |  |  |  |
| Number of metastasis>3 | 0.180 | 1.439 | 0.837 | 2.582 |  |  |  |  |
| Pulmonary metastasis | 0.234 | 0.701 | 0.390 | 1.259 |  |  |  |  |
| Metastatic organs>2 | 0.843 | 0.889 | 0.277 | 2.850 |  |  |  |  |
| HBV-DNA load | 0.332 | 1.361 | 0.731 | 2.533 |  |  |  |  |
| AFP>400 | 0.149 | 1.376 | 0.874 | 2.441 |  |  |  |  |
| TB>17.1 | 0.461 | 1.232 | 0.708 | 2.144 |  |  |  |  |
| ALT>44 | 0.740 | 0.891 | 0.450 | 1.763 |  |  |  |  |
| AST>44 | 0.717 | 1.114 | 0.621 | 2.000 |  |  |  |  |
| WBC>4 | 0.850 | 0.948 | 0.544 | 1.652 |  |  |  |  |
| PLT>100 | 0.302 | 1.361 | 0.758 | 2.443 |  |  |  |  |
| ALBI | 0.420 | 1.225 | 0.748 | 2.008 |  |  |  |  |
| Targeted therapy and /or Immunotherapy beore | 0.629 | 0.851 | 0.441 | 1.640 |  |  |  |  |

Table S2.

Univariate and Multivariable Analysis of DFS

| Variables |  | | Univariate Analysis | | |  | | Multivariable Analysis | | |
| --- | --- | --- | --- | --- | --- | --- | --- | --- | --- | --- |
|  | p | HR | | 95%CI | | p | HR | | 95%CI | |
| Folfox-placebo | 0.027 | 1.579 | | 1.053 | 2.368 | 0.037 | 1.541 | | 1.026 | 2.315 |
| Sex | 0.130 | 1.541 | | 0.881 | 2.696 |  |  | |  |  |
| Age | 0.162 | 1.333 | | 0.891 | 1.996 |  |  | |  |  |
| PVTT | 0.036 | 1.586 | | 1.031 | 2.441 | 0.833 | 0.925 | | 0.446 | 1.917 |
| T stage | 0.008 | 1.175 | | 1.044 | 1.324 | 0.093 | 1.187 | | 0.971 | 1.452 |
| N stage | 0.917 | 1.037 | | 0.521 | 2.067 |  |  | |  |  |
| Number of metastasis>3 | 0.100 | 1.442 | | 0.932 | 2.230 |  |  | |  |  |
| Pulmonary metastasis | 0.620 | 0.882 | | 0.539 | 1.446 |  |  | |  |  |
| Metastatic organs>2 | 0.883 | 0.939 | | 0.409 | 2.157 |  |  | |  |  |
| HBV-DNA load | 0.480 | 1.181 | | 0.744 | 1.874 |  |  | |  |  |
| AFP>400 | 0.073 | 1.448 | | 0.966 | 2.170 |  |  | |  |  |
| TB>17.1 | 0.814 | 1.057 | | 0.669 | 1.669 |  |  | |  |  |
| ALT>44 | 0.419 | 0.796 | | 0.457 | 1.385 |  |  | |  |  |
| AST>44 | 0.445 | 1.195 | | 0.756 | 1.889 |  |  | |  |  |
| WBC>4 | 0.079 | 0.678 | | 0.440 | 1.046 |  |  | |  |  |
| PLT>100 | 0.850 | 1.004 | | 0.671 | 1.622 |  |  | |  |  |
| ALBI | 0.527 | 1.130 | | 0.773 | 1.653 |  |  | |  |  |
| Targeted therapy and /or Immunotherapy beore | 0.549 | 1.155 | | 0.721 | 1.851 |  |  | |  |  |

Final protocol

**Combined FOLFOX4 with All-Trans Retinoic Acid versus FOLFOX4 with placebo in treatment of Advanced Hepatocellular Carcinoma with Extrahepatic Metastasis: A randomized, double blind comparative study (“ATFOX” study)**

# Introduction and Study Rationale

Hepatocellular carcinoma (HCC) is the fifth most common cancer in men, the eighth in women, and the third most frequent cause of cancer-related death worldwide, with more than 626,000 new cases per year^1^. The geographic distribution is not uniform across the world: approximately 80% of cases arise in Asia and Africa where HCC incidence is 30-120/100,000 in males, 9-30/100,000 in females. The incidence in Europe and North America States is < 5/100,000 in males, < 3/100,000 in females and is increasing in the recent three decades as a result of the high prevalence of Hepatitis C^2-4^. HCC commonly develops in a setting of chronic liver cell injury, which leads to inflammation, hepatocyte regeneration, liver matrix remodeling, fibrosis, and ultimately, cirrhosis^5 6^. The major etiologies of liver cirrhosis include chronic hepatitis B virus (HBV) and hepatitis C virus (HCV) infection, alcohol consumption, steatosis, aflatoxin exposure, diabetes, etc. In Asia, HCC is mainly due to chronic HBV infection^5^. Overall, HCC is associated with cirrhosis in about 80% of cases and is currently the leading cause of death among cirrhotic patients^6^.

In order to determine whether locally advanced, metastatic or recurrent HCCs are candidates of systemic treatment, in the last thirty years, many anti-cancer agents or combinations of agents have been tested in HCC, including systemic chemotherapy, immunotherapy and hormonal therapy^7-9^. The clinical application of more aggressive systemic chemotherapy regimens is severely limited by liver cirrhosis and compromised liver function in advanced stage HCC patients. Systematic reviews and meta-analyses consistently demonstrated that systemic chemotherapy did not prolong survival in patients with advanced HCC. The median overall survival (mOS) ranged from 3-7 months in most clinical studies, regardless of different etiology, ethnic group, and standard of care across the regions^7 10 11^.

Two recent randomized Phase III studies in HCC further confirmed limited effectiveness of chemotherapy in advanced HCC. A study conducted in Hong Kong testing doxorubicin versus PIAF (cisplatin, interferon α-2b, fluorouracil, and doxorubicin) combination showed no significant survival benefit (mOS: 8.6 vs 6.83 months, not statistically significant)^12^.Another international study of nolatrexed versus doxorubicin showed less survival benefit of nolatrexed compared to doxorubicin (mOS: 5.2 vs 7.53 months, p< .05)^13^.

Qin et al^14^ conducted a multicenter, open-label, randomized, phase III study in mainland China, Taiwan, Korea, and Thailand involved 371 patients age 18 to 75 years who had locally advanced or metastatic HCC and were ineligible for curative resection or local treatment. They were randomly assigned at a ratio of one to one to receive either FOLFOX4 (n=184) or doxorubicin (n= 187). Although the study did not meet its primary endpoint, the trend toward improved overall survival (OS) with FOLFOX, along with increased progression free survival (PFS) and response rate (RR), suggests that this regimen may confer some benefit to Asian patients, but an OS benefit cannot be concluded from these data^14^.

All-Trans Retinoic Acid (ATRA), a group of structural and functional analogues of vitamin A, exert fundamental effects on the regulation of epithelial cell growth, differentiation, and development^15^. ATRA exert their biological functions primarily by regulating gene expression through two distinct nuclear receptors, retinoic acid receptors and retinoid X receptors, which are both composed of three subtypes (α, β, and γ). Many of ATRA activities on the cellular level have been well characterized and translated to the regulation of processes like differentiation and cell death, which play critical roles in the outcome of malignant transformation of tissues. It is well known that ATRA is one of the strongest and the most thoroughly studied differentiation inducers which could induce differentiation of several types of tumor cells including stem cells. In fact, retinoid-based differentiation therapy of acute promyelocytic leukemia was one of the first successful examples of molecularly targeted treatment strategies. Our previous studies showed that ATRA could induce differentiation of HCC TICs via TS2/AKT pathway and combined treatment with ATRA and cisplatin would improve therapeutic effect, due to elimination of TICs via ATRA-induced differentiation in vivo and in vitro^16 17^. Thus, ATRA could be able to benefit both cancer prevention and cancer treatment in combination with classical chemotherapeutic agents ^18^. So far, there is no report regarding the efficacy and safety of ATRA and FOLFOX in the treatment of extrahepatic metastatic HCCs. Therefore, our hypothesis is that ATRA administration together with FOLFOX will prolong significant survival benefit compared to FOLFOX alone as palliative chemotherapy in advanced hepatocellular carcinoma patients with extrahepatic metastasis.

# Study Objectives

1. To determine whether ATRA + FOLFOX can improve the overall survival, progress-free survival, time to progress, symptomatic progress, and health-related quality of life.
2. To determine the safety of ATRA in the combination with FOLFOX.

# Study Design

This is a multicenter, double-blinded, placebo-controlled, randomized clinical trial in advanced HCC patients with extrahepatic metastasis. Subjects will be randomly assigned to one of the two arms, either ATRA+FOLFOX or FOLFOX alone. Our study has been registered at <http://www.chictr.org.cn> (ID: ChiCTR-IIR-17012916).

**
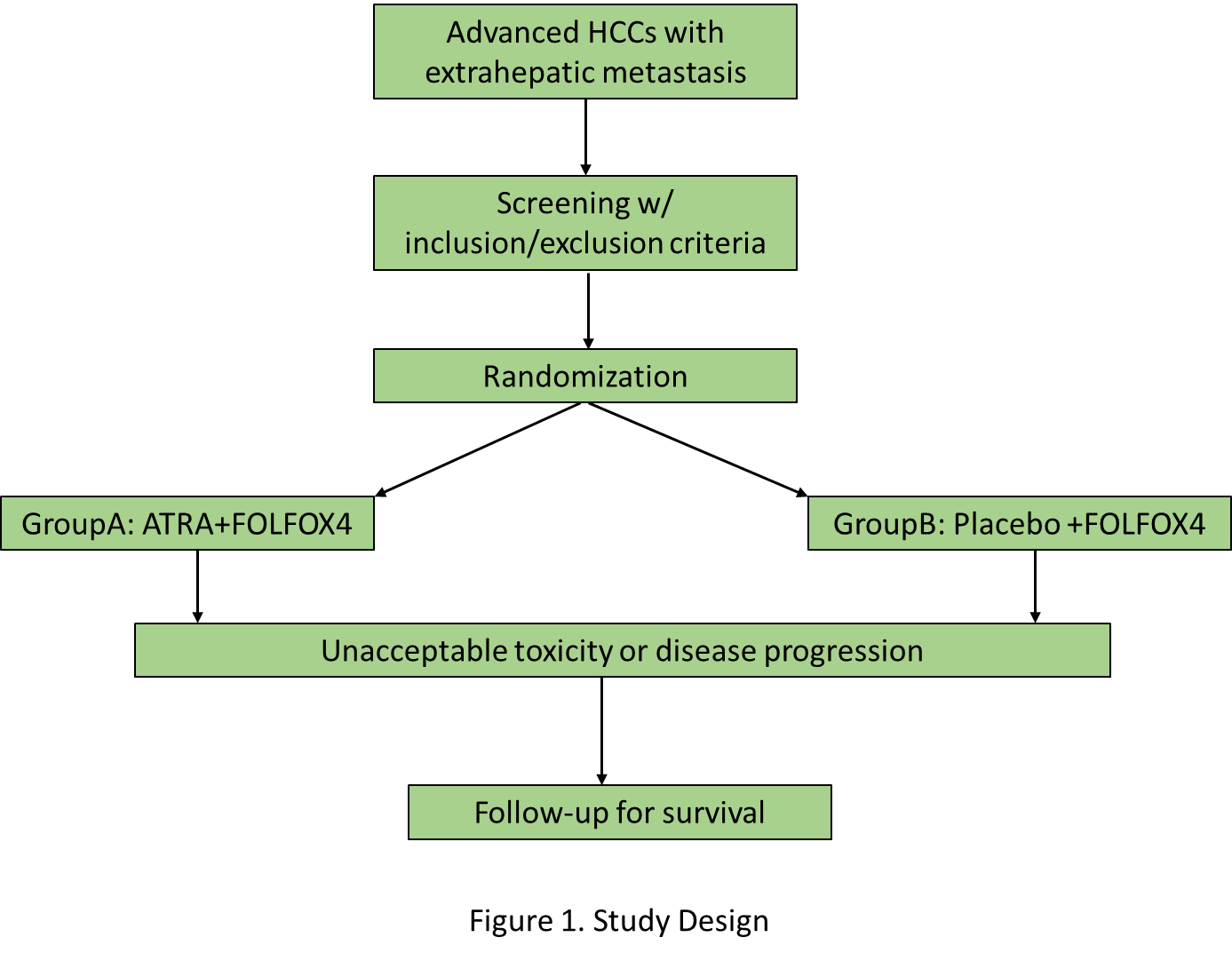
**

After screening for eligibility and signing of informed consent, qualified subjects will be randomized in a 1:1 ratio to receive ATRA+FOLFOX4 (Group A) or Placebo + FOLFOX4 (Group B). Randomization will be stratified by site and treatment assignments will remain blinded throughout the study.

Names of Therapeutic Agents:

- All-trans-Retinoic Acid (ATRA)
- Oxaliplatin (OXA)
- 5-Fluorouracil (5FU)
- Leucovorin (LV)

Mode of Administration:

ATRA+FOLFOX4 (Group A): ATRA 20 mg oral 3 times/day for 3 days prior to the initiation of chemo (FOLFOX4). ATRA will be discontinued at the end of FOLFOX4(OXA 85mg/m2 iv day1, LV 200mg/m2 iv day1 and day 2, 5FU 400mg/m2 iv bolus at hour 2 and then 600 mg/m2 iv over 22 hours on day 1 and day 2, once every 2 weeks) and a total of 6 cycles were performed. Placebo+FOLFOX4 (Group B):The placebo (replacing ATRA) will be given orally 3 times/day for 3 days prior to the chemo. The placebo will then be discontinued at the end of FOLFOX4 and a total of 6 cycles were performed. The follow-up phase began once a patient terminated the treatment phase.

Subjects will be evaluated for tumor response every 4 weeks. Tumor imagine evaluation will be performed by either computerized tomography (CT) scan or MRI. Progression will be determined based on modified RECIST criteria for HCC. Drug therapy will be continued until unacceptable toxicity or disease progression occurs. If investigator determines the subject is benefiting from the blinded study drug and the subject is willing to continue the blinded study drug, the treatment may be continued beyond radiographic progression. Choice of subsequent therapies in case of progressive disease will be at the best discretion of the investigator, which should be beneficial to subjects overall. Subsequent treatments for HCC will be collected in the follow-up period. Requests for un-blinding will only be granted for emergency medical management. Subjects who have discontinued study therapy due to study drug toxicity or any reason other than confirmed tumor progression will continue to have tumor assessments every 4 weeks until documented radiographic tumor progression. All randomized subjects will be followed for overall survival until the required number of events has been reached.

Discontinuation of Subjects from Treatment: subjects must discontinue the investigational agents for any of the following reasons:

1. Withdrawal of informed consent (subject’s decision to withdraw for any reason).
2. Any clinical adverse event (AE), laboratory abnormality or intercurrent illness which,
3. in the opinion of the investigator, indicates that continued participation in the study is not in the best interest of the subject.
4. Pregnancy.
5. Loss of ability to freely provide consent due to either a psychiatric or physical (e.g. infectious disease) illness.
6. Disease progression*

* Note: If investigator determines the subject is benefiting from the blinded study drug (for example: subject may be experiencing symptomatic improvement at the time of documented radiographic progression) and the subject is willing to continue the blinded study drug, the treatment may be continued beyond radiographic progression.

# Standard Best Supportive Care

All subjects enrolled in this clinical study should always receive Standard Best Supportive Care (SBSC). SBSC is defined by the institutional standards of the participating clinical centers. In general, SBSC has to be understood as the multi-professional attention to the patient’s overall physical, psychosocial, spiritual, and cultural needs available at all stages of the illness. Regarding the physical well-being of HCC subjects enrolled in this trial, SBSC may include but may not be restricted to: (i) blood transfusion for anemic state; (ii) antibiotics to control infections; (iii) analgesics, including non-steroidal anti-inflammatory drugs, opioids, and corticosteroids; (iv) antiemetic drugs; (v) vitamin and nutritional support and; (vi) alternative medicine (include herbal medicine) are allowed except of those have any statement of "anticancer activity" or "immunologic activity" in the labels. Localized radiation therapy to alleviate symptoms such as pain due to non-target lesions is allowed according to institutional standards. Other palliative care will be also provided to the best interest of a subject is receiving study drug.

# Study Population

Pathologically confirmed hepatocellular carcinoma with extrahepatic metastasis at the Eastern Hepatobilliary Surgery Hospital, Zhejiang Sian Hospital and Fujian Provincial Cancer Hospital.

## 5.1 Inclusion Criteria

1. Male and female, ages 18-75 and above, with documented advanced HCC
2. Child-Pugh Class A.
3. With confirmed extrahepatic metastatic lesions located in bone, lung, abdominal metastasis including lymph nodes.
4. ECOG performance status Grade 1 or less.
5. Liver and renal lab test must meet the following ranges:
   1. Total bilirubin equal or less than 1.5 times of upper normal limit (UNL) defined by each clinical lab in each site.
   2. ALT and AST less than 2.5 times of times of upper normal limit (UNL) defined by each clinical lab in each site.
   3. Serum Creatinine equal or less than 1 times of upper normal limit (UNL) defined by each clinical lab in each site.
   4. Endogenous creatinine clearance rate >50ml/min (calculated with Cockcroft-Gault formulation).
6. Other lab tests:
   1. Hemoglobin >= 90g/L
   2. Platelet Count >=75 ˟10 ^9^/L
   3. WBC Count >=3.5 ˟10 ^9^/L
7. Good cardiac function, no myocardial infarction within 6 months; well controlled hypertension and coronary heart disease if any.

## 5.2 Exclusion Criteria

1. No extrahepatic metastatic lesion found.
2. Decompensated liver function or hepatic encephalopathy.
3. Pregnant or breast-feeding women.
4. Uncontrolled hypertension even with medication.
5. Patients with hemorrhagic disorders or active bleeding or sepsis
6. History of HIV infection and AIDS or other acute or chronic disease.
7. Active or uncontrolled severe infections.
8. CHF NYHA class > 2, active CAD or cardiac ischemia, uncontrolled cardiac arrhythmias even with anti-arrhythmic therapy.
9. Unable to take oral medication.
10. Low compliance judged by investigators.
11. Seizure disorder requiring medication (such as steroids or anti-epileptics).
12. History of drug abuse, or mental disorders which could compromise compliance.
13. Any other conduction which may jeopardize patient safety and compliance.

# Informed Consent

The Investigator must ensure that written informed consent to participate in the study is obtained before including any individual as a subject in the study, and before conducting any study-related assessments. The investigator must provide the prospective subject, or the prospective subject’s legally authorized representative, with sufficient opportunity to consider whether to participate, and minimize the possibility of coercion or undue influence. The process is designed to 1) give the subject all the information that he/she needs, 2) ensure that the subject understands the information and 3) give the subject a chance to consider study participation. The process should permit the subject to ask questions and exchange information freely.

Specifically, the Investigator is to explain to each subject all elements of informed consent as specified by CFDA. This also includes explaining that photographs will be taken and may be used in publications in ways that do not identify the subject. After the explanation, the subject or legally authorized representative will voluntarily sign and date the consent/assent form if they wish to participate in the study. A copy of the consent/ assent form must be provided to the subject or the subject’s legally authorized representative. A signed and dated copy of the consent/assent form must be always maintained in the Investigator Site File. The informed consent process must be followed, and the subject’s participation in the study, must be documented in the subject’s medical record/chart.

# Endpoints and Assessment

## 7.1 Primary Endpoint

Overall Survival (OS): OS is defined as the time from randomization to the time of death from any cause. Subjects who are alive at the time of the final analysis or who have become lost to follow-up will be censored at their last known alive date.

## 7.2 Secondary Endpoints

Progression Free Survival (PFS): PFS is defined as the time (in months) from randomization to the time of radiographic disease progression. Subjects without progression will be censored at their last tumor assessment date. Subjects who have no on-study tumor assessments will be censored at the date of randomization.

Objective Response Rate (ORR): ORR is defined as the proportion of randomized subjects in each treatment arm whose best response is a CR or PR using the modified RECIST for HCC.

Disease Control Rate (DCR): DCR is defined as the proportion of randomized subjects in each treatment arm whose best response is a CR, PR or SD using the modified RECIST for HCC.

## 7.3 Safety Endpoints

Study drug toxicities will be assessed continuously, which include vital sign, physical exam, neurological exam, EKG, Echo, clinical lab tests (hemoglobin, hematocrit, red blood cell, total leukocyte count with differential, platelet count, AST, ALT, total bilirubin, direct bilirubin, alkaline phosphatase, lactate dehydrogenase (LDH), creatinine, BUN or urea, blood glucose, total protein, albumin, sodium, potassium, chloride, total calcium, phosphorus, magnesium, ammonia, and AFP) and specialty exams (chest X-ray, CT, MRI, Bone Scan, etc.). All the adverse events will be evaluated according to the NCI CTCAE (Version 4.0), on a continuous basis while the subject is on study. Scheduled evaluations will occur every 4 weeks.

| **Parameters** | **Definitions** |
| --- | --- |
| **Complete Response (CR)** | Complete disappearance of extrahepatic metastatic lesions under the contrast-agent enhancement in the arterial phase of spiral CT or MRI. CR must be confirmed by a second evaluation no less than 4 weeks after the date the CR was first obtained. |
| **Partial Response (PR)** | Decrease of > 30% in the sum of the longest diameters (SLD) of extrahepatic metastatic lesions taking reference the baseline SLD. PR must be confirmed by a second evaluation no less than 4 weeks after the date the PR was first obtained. |
| **Stable Disease (SD)** | Failure to meet criteria for complete or partial response, in the absence of progressive disease. |
| **Progressive Disease (PD)** | - Increase of > 20% in the SLD of extrahepatic metastatic lesions taking reference the smallest SLD of target lesions recorded since the treatment started. If the 20% or more increase is observed in two consecutive determinations, the date of PD is the date of the first evaluation. OR - New extrahepatic metastatic lesions with the longest diameter of at least 10 mm confirmed by contrast-enhanced spiral CT or MRI imaging. OR - Appearance of one or more new extrahepatic lesions of any size. OR - Appearance of more intrahepatic lesions, expanding existing lesions (including portal vein emboli). |
|  |  |

# Safety Reporting

## 8.1 Adverse Events (AEs)

An Adverse Event (AE) is defined as any new untoward medical occurrence or worsening of a pre-existing medical condition in a patient or clinical investigation subject administered an investigational product and that does not necessarily have a causal relationship with this treatment. An AE can therefore be any unfavorable and unintended sign (including an abnormal laboratory finding, for example), symptom, or disease temporally associated with the use of investigational product, whether or not considered related to the investigational product.

## 8.2 Serious Adverse Events (SAEs)

SAEs are any of the following untoward medical events:

- Death.
- Life-threatening (defined as an event in which the subject was at risk of death.
- Inpatient hospitalization or causes prolongation of existing hospitalization.
- Persistent or significant disability/incapacity.
- Congenital anomaly/birth defects.

## 8.3 Judgement of AEs/SAEs

All AEs and SAEs must be graded according to the National Cancer CTCAE version no. 3, 09-Aug-2006. The following categories and definitions of causal relationship to study drugs as determined by a physician:

- Related: There is a reasonable causal relationship to study drug administration and AEs and SAEs
- Not Related: There is not a reasonable causal relationship to study drug administration and the AE and SAEs. The expression “reasonable causal relationship” is meant to convey in general that there are facts or other arguments to suggest a positive causal relationship.

## 8.4 Collection and Reporting

Adverse events can be spontaneously reported or elicited during open-ended questioning, examination, or evaluation of a subject (to prevent reporting bias, subjects should not be questioned regarding the specific occurrence of one or more AEs).

If known, the diagnosis of the underlying illness or disorder should be recorded, rather than its individual symptoms. The following information should be captured for all AEs: onset, duration, intensity, seriousness, relationship to study drug, action taken, and treatment required. If treatment for the AE was administered, it should be recorded on the appropriate CRF page. The investigator shall supply the sponsor and Ethics Committee with any additional requested information, notably for reported deaths of subjects. Completion of supplemental CRFs may be requested for AEs and/or laboratory abnormalities that are reported/identified during the course of the study.

# Statistical Considerations

## 9.1 Sample Size Estimation

The sample size is calculated to compare overall survival (OS) between two arms. The sample size was estimated on the basis of the overall survival (OS) of 14.9 mon in the combined group and 8.2 mon in the FOLFOX4 only group, which were obtained from the data of our retrospective cohort of patients. The minimum sample size was 106 for the two groups (two-sided α= 0.05; β = 0.20; power, 80%;10% of patients were added to compensate for any loss to follow-up).


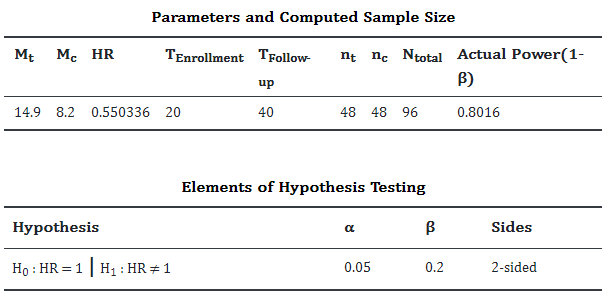


**9.2 Populations for Analysis**

Intent-to-treat (ITT) population: any patients assigned to a treatment group by the randomization process, regardless of whether patients received any study treatment or received a different study treatment from which they were randomized. This is the primary data set for the test of overall survival, analyses of secondary efficacy endpoints like TTP, symptom assessment and baseline characteristics.

Per protocol (PP) population: all randomized subjects except for subjects who 1) have wrong diagnosis of cancer; 2) are not treated; 3) are not treated with the study therapy as assigned by the randomization. This is the primary dataset for the test of overall survival.

Safety population: All subjects who received at least one dose of study medications. This is the primary data set for dosing, safety, concomitant medications.

## 9.3 Analysis of Efficacy

OS will be compared between two treatment groups, using a log-rank test procedure at 5% significance level and the survival curves will be estimated using Kaplan-Meier estimates. Additional analyses of survival will include the computation of hazard ratios (HRs) and the estimation of survival functions. The survival hazard ratio of TARA+FOLFOX to placebo + FOLFOX and an associated two-sided 95.0% confidence interval will be computed using unadjusted and adjusted Cox proportional hazards modeling. Cox models will include treatment stratified by the above-mentioned stratification factors, and an adjusted model which will include a pre-defined list of covariates (next paragraph) as well as the above-mentioned stratification factors, all as covariates. The response rater (CR and PR) will be compared between two treatments using Chi-Square Test or Fisher’s Exact Test. Repeated measures ANOVA (if applicable) will be used or two-group t-test/Wilcoxon signed rank test on difference between baseline score and the best score in each scale will be used to compare quality of life between two treatments.

## 9.4 Analysis of Safety

We will conduct a safety analysis on the safety population. Worst toxicity grades per subject will be tabulated for selected AEs and laboratory measurements. All recorded AEs, SAEs and AEs leading to study therapy discontinuation will be listed and tabulated. Vital signs and clinical laboratory test results will be listed and summarized by treatment arms. Any significant physical examination findings, ECG results, and clinical laboratory results will be listed.

# Schedule of Events

| **Visits** | **Enrollment & Randomization** | **M1** | **M2** | **M3** | **M4** | **M5** | **M6** | **M7** | **M8** | **M9** | **M10** | **M11** | **M12** |
| --- | --- | --- | --- | --- | --- | --- | --- | --- | --- | --- | --- | --- | --- |
| **Identification** | X |  |  |  |  |  |  |  |  |  |  |  |  |
| **Screen by inclusion/exclusion criteria** | X |  |  |  |  |  |  |  |  |  |  |  |  |
| **Informed Consent Form** | X |  |  |  |  |  |  |  |  |  |  |  |  |
| **Initial Assessment (history/physical/lab/imaging)** | X |  |  |  |  |  |  |  |  |  |  |  |  |
| **Assessment – History/Physical** | X | X | X | X | X | X | X | X | X | X | X | X | X |
| **CT Scan/MRI** | X | X | X | X | X | X | X | X | X | X | X | X | X |
| **Laboratory Tests** | X | X | X | X | X | X | X | X | X | X | X | X | X |
| **Quality of life Questionnaires** | X | X | X | X | X | X | X | X | X | X | X | X | X |
| **Overall Survival** |  | X | X | X | X | X | X | X | X | X | X | X | X |
| **Tumor Progression Assessment** |  | X | X | X | X | X | X | X | X | X | X | X | X |
| **Concomitant Medication** | X | X | X | X | X | X | X | X | X | X | X | X | X |
| **AEs/SAEs** |  | X | X | X | X | X | X | X | X | X | X | X | X |

**11. Discussion**

85% of HCC patients present with an advanced disease stage at diagnosis, and a large number of patients diagnosed with early-stage disease eventually experience recurrence**^23^**. Sorafenib is the only drug approved by FDA that extends the overall survival time of patients with advanced HCC by approximately three months in the Barcelona Clinic Liver Cancer (BCLC) staging classification. The response rate is only 2-3% and the acquisition of a CR was rarer**^24^**. In contrast to other solid cancer, chemotherapy has not been used routinely in HCC before because of the toxicity and high incidence of chemo-resistance.

However, with the constant progress of chemotherapeutics especially platinum drugs, recent trials have proved that chemotherapy may have a role in the treatment of advanced HCC. In one recent study**^25^**, metronomic chemotherapy including 5-FU and cisplatin was associated with more favorable outcomes in terms of overall survival than was sorafenib in advanced HCC (158 days vs. 117 days, p=0.029). In the EACH study**^14^**, FOLFOX showed an improvement in median overall survival and similar rate of AEs compared with doxorubicin (6.40 months vs 4.97 months, p=0.07), and has been established as an option in the treatment of advanced HCC.

Despite the advances in systemic chemotherapy, drug resistance is still an obstacle to acquire further improvement. Increasing evidence has shown that tumor initiating cells (TICs) exhibit greater resistance to conventional chemotherapies than non-TICs. It is well known that ATRA is one of the strongest and the most thoroughly studied differentiation inducers which could induce differentiation of kinds of tumor cells including stem cells. Our previous studies**^16,17^** showed that ATRA could induce differentiation of HCC TICs via TS2/AKT pathway and combined treatment with ATRA and Cisplatin could improve therapeutic effect, due to elimination of TICs via ATRA-induced differentiation in vivo and in vitro.  Therefore, our hypothesis holds that a ATRA/FOLFOX to the extrahepatic metastasis might be useful and no prospective trial has been investigated in this setting so far.

**References**

1. Kamangar F, Dores GM, Anderson WF. Patterns of cancer incidence, mortality, and prevalence across five continents: defining priorities to reduce cancer disparities in different geographic regions of the world. J Clin Oncol 2006;24(14):2137-50. doi: 10.1200/JCO.2005.05.2308

2. El-Serag HB, Mason AC. Rising incidence of hepatocellular carcinoma in the United States. N Engl J Med 1999;340(10):745-50. doi: 10.1056/NEJM199903113401001

3. Sherman M. Hepatocellular carcinoma: epidemiology, risk factors, and screening. Semin Liver Dis 2005;25(2):143-54. doi: 10.1055/s-2005-871194

4. Thomas MB, Zhu AX. Hepatocellular carcinoma: the need for progress. J Clin Oncol 2005;23(13):2892-9. doi: 10.1200/JCO.2005.03.196

5. Okuda K. Hepatocellular carcinoma: recent progress. Hepatology 1992;15(5):948-63.

6. Sangiovanni A, Del Ninno E, Fasani P, et al. Increased survival of cirrhotic patients with a hepatocellular carcinoma detected during surveillance. Gastroenterology 2004;126(4):1005-14.

7. Johnson PJ. Systemic chemotherapy of liver tumors. Semin Surg Oncol 2000;19(2):116-24.

8. Llovet JM, Sala M. Non-surgical therapies of hepatocellular carcinoma. Eur J Gastroenterol Hepatol 2005;17(5):505-13.

9. Llovet JM. Updated treatment approach to hepatocellular carcinoma. J Gastroenterol 2005;40(3):225-35. doi: 10.1007/s00535-005-1566-3

10. Simonetti RG, Liberati A, Angiolini C, et al. Treatment of hepatocellular carcinoma: a systematic review of randomized controlled trials. Ann Oncol 1997;8(2):117-36.

11. Mathurin P, Rixe O, Carbonell N, et al. Review article: Overview of medical treatments in unresectable hepatocellular carcinoma--an impossible meta-analysis? Aliment Pharmacol Ther 1998;12(2):111-26.

12. Yeo W, Mok TS, Zee B, et al. A randomized phase III study of doxorubicin versus cisplatin/interferon alpha-2b/doxorubicin/fluorouracil (PIAF) combination chemotherapy for unresectable hepatocellular carcinoma. J Natl Cancer Inst 2005;97(20):1532-8. doi: 10.1093/jnci/dji315

13. Gish RG, Porta C, Lazar L, et al. Phase III randomized controlled trial comparing the survival of patients with unresectable hepatocellular carcinoma treated with nolatrexed or doxorubicin. J Clin Oncol 2007;25(21):3069-75. doi: 10.1200/JCO.2006.08.4046

14. Qin S, Bai Y, Lim HY, et al. Randomized, multicenter, open-label study of oxaliplatin plus fluorouracil/leucovorin versus doxorubicin as palliative chemotherapy in patients with advanced hepatocellular carcinoma from Asia. J Clin Oncol 2013;31(28):3501-8. doi: 10.1200/JCO.2012.44.5643

15. Uray IP, Dmitrovsky E, Brown PH. Retinoids and rexinoids in cancer prevention: from laboratory to clinic. Semin Oncol 2016;43(1):49-64. doi: 10.1053/j.seminoncol.2015.09.002

16. Zhang Y, Guan DX, Shi J, et al. All-trans retinoic acid potentiates the chemotherapeutic effect of cisplatin by inducing differentiation of tumor initiating cells in liver cancer. J Hepatol 2013;59(6):1255-63. doi: 10.1016/j.jhep.2013.07.009

17. Guan DX, Shi J, Zhang Y, et al. Sorafenib enriches epithelial cell adhesion molecule-positive tumor initiating cells and exacerbates a subtype of hepatocellular carcinoma through TSC2-AKT cascade. Hepatology 2015;62(6):1791-803. doi: 10.1002/hep.28117

18. Tang XH, Gudas LJ. Retinoids, retinoic acid receptors, and cancer. Annu Rev Pathol 2011;6:345-64. doi: 10.1146/annurev-pathol-011110-130303

19. Luckett T, King MT, Butow PN, et al. Choosing between the EORTC QLQ-C30 and FACT-G for measuring health-related quality of life in cancer clinical research: issues, evidence and recommendations. Ann Oncol 2011;22(10):2179-90. doi: 10.1093/annonc/mdq721

20. Smith AB, Cocks K, Parry D, et al. Reporting of health-related quality of life (HRQOL) data in oncology trials: a comparison of the European Organization for Research and Treatment of Cancer Quality of Life (EORTC QLQ-C30) and the Functional Assessment of Cancer Therapy-General (FACT-G). Qual Life Res 2014;23(3):971-6. doi: 10.1007/s11136-013-0534-2

21. Gandhi S, Khubchandani S, Iyer R. Quality of life and hepatocellular carcinoma. J Gastrointest Oncol 2014;5(4):296-317. doi: 10.3978/j.issn.2078-6891.2014.046

22. Li L, Yeo W. Value of quality of life analysis in liver cancer: A clinician's perspective. World J Hepatol 2017;9(20):867-83. doi: 10.4254/wjh.v9.i20.867

23. Roxburgh P, Evans TR. Systemic therapy of hepatocellular carcinoma: are we making progress? Adv Ther 2008;25:1089-104.

24. Llovet JM, Ricci S, Mazzaferro V, et al; SHARP Investigators Study Group. Sorafenib in advanced hepatocellular carcinoma. N Engl J Med 2008; 359:378-90.

25. Shiozawa K, Watanabe M, Ikehara T,et al.Sustained complete response of hepatocellular carcinoma with portal vein tumor thrombus following discontinuation of sorafenib: A case report. Oncol Lett. 2014;7(1):50-52.

SAP

Statistical Analysis Plan (SAP) for AT-FOX analysis

**1. Study Background**

**1.1 Study Objective**

The presence of extrahepatic metastasis (EHM) is a major cause of the death of hepatocellular carcinoma (HCC) patients, but there is no standard treatment and even no prospective clinical trials have even been designed specifically for HCC with EHM till now. Systemic treatment is the only recommended therapy according to the BCLC staging system and the Chinese Guidelines for The Diagnosis and Treatment of Hepatocellular Carcinoma. But the expected median survival time was only 6 to 10 months. All-Trans Retinoic Acid (ATRA) is known as one of the strongest differentiation inducers which could induce differentiation of several types of tumor cells including stem cells. Our previously studies showed that ATRA potentiates the chemotherapeutic effect of cisplatin/oxaliplatin. Our retrospective clinical studies also confirmed the efficacy of combination therapy which could prolong the median OS to 14.0 month.

Therefore, the primary objective of this trial is to study the effect of ATRA-FOLFOX4 on the efficacy of treatment of HCC with EHM.

**1.2 Primary hypothesis**

The primary hypothesis of the project is that ATRA-FOLFOX4 leads to a better overall survival compared to FOLFOX4 alone.

**1.3 Secondary hypothesis**

- ATRA-FOLFOX4 leads in HCC patients with EHM compared to FOLFOX4 alone to better progress-free survival.
- To determine the safety of ATRA in the combination with FOLFOX

**1.4 Study design**

This is a multicenter, double-blinded, placebo-controlled, randomized clinical trial in advanced HCC patients with EHM. The randomization schedule was generated by an independent statistician using randomization block method in SAS 9.4. Eligible subjects enrolled in the study all received a unique subject identification number and were randomly assigned to a treatment group ATRA- FOLFOX4 or FOLFOX4 alone in a ratio of 1:1 (Website: http://pro32.irtone.com/). Both subjects and investigators did not know the true treatments and were blinded until emergency un-blinding and after database lock. Our study has been registered at <http://www.chictr.org.cn> (ID: ChiCTR-IIR-17012916).

After screening for eligibility and signing of informed consent, qualified subjects will be randomized in a 1:1 ratio to receive ATRA+FOLFOX4 (Group A) or Placebo + FOLFOX4 (Group B). Randomization will be stratified by site and treatment assignments will remain blinded throughout the study.

Names of Therapeutic Agents:

- All-trans-Retinoic Acid (ATRA)
- Oxaliplatin (OXA)
- 5-Fluorouracil (5FU)
- Leucovorin (LV)

Mode of Administration:

ATRA+FOLFOX4 (Group A): ATRA 20 mg oral 3 times/day for 3 days prior to the initiation of chemo (FOLFOX4). ATRA will be discontinued at the end of FOLFOX4(OXA 85mg/m2 iv day1, LV 200mg/m2 iv day1 and day 2, 5FU 400mg/m2 iv bolus at hour 2 and then 600 mg/m2 iv over 22 hours on day 1 and day 2, once every 2 weeks) and a total of 6 cycles were performed. Placebo+FOLFOX4 (Group B):The placebo (replacing ATRA) will be given orally 3 times/day for 3 days prior to the chemo. The placebo will then be discontinued at the end of FOLFOX4 and a total of 6 cycles were performed. The follow-up phase began once a patient terminated the treatment phase.

A more detailed description of the in- and exclusion criteria can be found in the in- and exclusion criteria below.

**1.5 In- and exclusion criteria**

The treatment candidates were defined as having the following characteristics.

- General inclusion criteria:

Male and female, ages 18-75 and above, with documented advanced HCC; Child-Pugh Class A; With confirmed extrahepatic metastatic lesions located in lung, bone, abdominal metastasis including lymph nodes; ECOG performance status Grade 1 or less; life expectancy 3 months; Liver and renal lab test must meet the following ranges: Total bilirubin equal or less than 1.5 times of upper normal limit (UNL) defined by each clinical lab in each site, ALT and AST less than 2.5 times of times of upper normal limit (UNL) defined by each clinical lab in each site, Serum Creatinine equal or less than 1 times of upper normal limit (UNL) defined by each clinical lab in each site, Endogenous creatinine clearance rate >50ml/min (calculated with Cockcroft-Gault formulation). Other lab tests: Hemoglobin ≧ 90g/L, Platelet Count ≧75 ˟10 9/L, WBC Count ≧3.5 ˟10 9/L. Good cardiac function, no myocardial infarction within 6 months; well controlled hypertension and coronary heart disease if any.

- General inclusion criteria:

No extrahepatic metastatic lesion found; decompensated liver function or hepatic encephalopathy; egnant or breast-feeding women; ucontrolled hypertension even with medication; pients with hemorrhagic disorders or active bleeding or sepsis; history of HIV infection and AIDS or other acute or chronic disease; active or uncontrolled severe infections; CHF NYHA class > 2, active CAD or cardiac ischemia, uncontrolled cardiac arrhythmias even with anti-arrhythmic therapy; unable to take oral medication; low compliance judged by investigators; seizure disorder requiring medication (such as steroids or anti-epileptics); history of drug abuse, or mental disorders which could compromise compliance; any other conduction which may jeopardize patient safety and compliance.

**1.6 Sample Size Calculation**

The sample size is calculated to compare overall survival (OS) between two arms. The sample size was estimated on the basis of the overall survival (OS) of 14.9 mon in the combined group and 8.2 mon in the FOLFOX4 only group, which were obtained from the data of our retrospective cohort of patients. The minimum sample size was 106 for the two groups (two-sided α= 0.05; β = 0.20; power, 80%;10% of patients were added to compensate for any loss to follow-up).

**2. Analysis sets**

**2.1 Definitions**

The full analysis set comprises all subjects who were so called treatment candidates irrespective of the actually received treatment.

The per-protocol analysis set comprises the same treatment candidates, with the exclusion of those who were not treated by ATRA-FOLFOX4.

The safety analysis set: The safety analysis set is the same as the intention-to-treat analysis set without estimates for missing values.

Missing values that are assumed to be missings at random (MAR) or missings completely at random (MCAR) are estimated using multiple imputation methods for all analysis sets except analysis set.

**2.2 Application**

Analyses in the full analysis set: The intention-to-treat analysis, any patients assigned to a treatment group by the randomization process, regardless of whether patients received any study treatment or received a different study treatment from which they were randomized. This is the primary data set for the test of overall survival, analyses of secondary efficacy endpoints like TTP, symptom assessment and baseline characteristics.

Analyses in per-protocol analysis set: The per-protocol analysis, all randomized subjects except for subjects who 1) have wrong diagnosis of cancer; 2) are not treated; 3) are not treated with the study therapy as assigned by the randomization. This is the primary dataset for the test of overall survival.

Analyses in safety analysis set: All subjects who received at least one dose of study medications. This is the primary data set for dosing, safety, concomitant medications.

**3. Trial centers**

Data for these analyses were obtained from the ATFOX registry, which is registered under ChiCTR-IIR-17012916. Participating centers:

- Eastern Hepatobiliary Surgery Hospital
- Fujian Provincial Cancer Hospital
- Zhejiang Sian International Hospital

Datasets for final analysis database was locked and unblinded on 31**^nd^** Dec. 2021.

**4. Analysis variables**

**4.1 Primary outcome**

Overall Survival (OS): OS is defined as the time from randomization to the time of death from any cause. Subjects who are alive at the time of the final analysis or who have become lost to follow-up will be censored at their last known alive date.

**4.2 Secondary outcome**

Progression Free Survival (PFS): PFS is defined as the time (in months) from randomization to the time of radiographic disease progression. Subjects without progression will be censored at their last tumor assessment date. Subjects who have no on-study tumor assessments will be censored at the date of randomization.

Objective Response Rate (ORR): ORR is defined as the proportion of randomized subjects in each treatment arm whose best response is a CR or PR using the modified RECIST for HCC.

Disease Control Rate (DCR): DCR is defined as the proportion of randomized subjects in each treatment arm whose best response is a CR, PR or SD using the modified RECIST for HCC.

**4.3 Safety Outcomes**

Study drug toxicities will be assessed continuously, which include vital sign, physical exam, neurological exam, EKG, Echo, clinical lab tests (hemoglobin, hematocrit, red blood cell, total leukocyte count with differential, platelet count, AST, ALT, total bilirubin, direct bilirubin, alkaline phosphatase, lactate dehydrogenase (LDH), creatinine, BUN or urea, blood glucose, total protein, albumin, sodium, potassium, chloride, total calcium, phosphorus, magnesium, ammonia, and AFP) and specialty exams (chest X-ray, CT, MRI, Bone Scan, etc.). All the adverse events will be evaluated according to the NCI CTCAE (Version 4.0), on a continuous basis while the subject is on study. Scheduled evaluations will occur every 4 weeks.

**5. Statistical analyses**

For all analyses (including analysis of primary outcome) appropriate descriptive statistics (mean, standard deviation, median, interquartile range, percentages) depending on the scale and distribution of the outcome variable are presented by treatment group. Standardized mean differences are provided where possible in order to provide insights into the magnitude of differences between the treatment groups.

**5.1 Primary analyses**

OS will be compared between two treatment groups, using a log-rank test procedure at 5% significance level and the survival curves will be estimated using Kaplan-Meier estimates. Additional analyses of survival will include the computation of hazard ratios (HRs) and the estimation of survival functions. The survival hazard ratio of TARA+FOLFOX to placebo + FOLFOX and an associated two-sided 95.0% confidence interval will be computed using unadjusted and adjusted Cox proportional hazards modeling. Cox models will include treatment stratified by the above-mentioned stratification factors, and an adjusted model which will include a pre-defined list of covariates as well as the above-mentioned stratification factors, all as covariates. This analysis is executed based according to the intention-to-treat principle (ITT).

**5.2 Secondary analyses**

The secondary outcomes as mentioned under 4.2 are analyzed as follows:

PFS will be compared between two treatment groups, using a log-rank test procedure at 5% significance level and the survival curves will be estimated using Kaplan-Meier estimates. This analysis is executed based according to the intention-to-treat principle (ITT).

The efficiency (ORR and DCR) will be compared between two treatments using Chi-Square Test or Fisher’s Exact Test. Repeated measures ANOVA (if applicable) will be used or two-group t-test/Wilcoxon signed rank test on difference between baseline score. This analysis is executed based according to the intention-to-treat principle (ITT).

**5.3 Safety Analyses**

We report absolute frequencies of events as well as percentages per group separately by safety measure without imputed values for missing in the full analysis set.
